# Hepatic passaging of *NRAS*-mutant melanoma influences adhesive properties and metastatic pattern

**DOI:** 10.1186/s12885-023-10912-4

**Published:** 2023-05-13

**Authors:** Bianca Dietsch, Céline Weller, Carsten Sticht, Carolina de la Torre, Martin Kramer, Sergij Goerdt, Cyrill Géraud, Sebastian A. Wohlfeil

**Affiliations:** 1 Department of Dermatology, Venereology, and Allergology, University Medical Center and Medical Faculty Mannheim, Heidelberg University, and Center of Excellence in Dermatology, Mannheim, Germany; 2 Section of Clinical and Molecular Dermatology, Medical Faculty Mannheim, Heidelberg University, Mannheim, Germany; 3 NGS Core Facility, Medical Faculty Mannheim, Heidelberg University, Mannheim, Germany; 4 Department of Veterinary Clinical Sciences, Small Animal Clinic, Justus-Liebig-University Giessen, Giessen, Germany; 5 European Center for Angioscience, Medical Faculty Mannheim, Heidelberg University, Mannheim, Germany; 6 Skin Cancer Unit, German Cancer Research Center (DKFZ), Heidelberg, Germany

**Keywords:** Cutaneous melanoma, Melanoma metastasis, Liver metastasis, Tumor heterogeneity

## Abstract

**Background:**

Liver metastasis is a poor prognostic factor for treatment of advanced cutaneous melanoma with either immunotherapy or targeted therapies. In this study we focused on *NRAS* mutated melanoma, a cohort with high unmet clinical need.

**Methods:**

WT31 melanoma was repeatedly passaged over the liver after intravenous injections five times generating the subline WT31_P5IV. The colonization of target organs, morphology, vascularization and the gene expression profiles of metastases were analyzed.

**Results:**

After intravenous injection lung metastasis was significantly decreased and a trend towards increased liver metastasis was detected for WT31_P5IV as compared to parental WT31. Besides, the ratio of lung to liver metastases was significantly smaller. Histology of lung metastases revealed reduced proliferation of WT31_P5IV in relation to WT31 while both size and necrotic areas were unaltered. Liver metastases of both sublines showed no differences in vascularization, proliferation or necrosis. To identify tumor-intrinsic factors that altered the metastatic pattern of WT31_P5IV RNA sequencing was performed and revealed a differential regulation of pathways involved in cell adhesion. Ex vivo fluorescence imaging confirmed that initial tumor cell retention in the lungs was significantly reduced in WT31_P5IV in comparison to WT31.

**Conclusion:**

This study demonstrates that tumor-intrinsic properties influencing the metastatic pattern of *NRAS* mutated melanoma are strongly affected by hepatic passaging and the hematogenous route tumor cells take. It has implications for the clinical setting as such effects might also occur during metastatic spread or disease progression in melanoma patients.

**Supplementary Information:**

The online version contains supplementary material available at 10.1186/s12885-023-10912-4.

## Background

Cutaneous melanoma (CM) is a cancer that arises from melanocytes of the skin and preferentially spreads to the skin, lymph nodes, lung, liver and brain [1]. Metastatic disease is treated by immune checkpoint inhibition (ICI) or targeted therapies (TT). However, around two thirds to half of stage IV melanoma patients suffer a relapse. Especially, liver metastasis is described as negative predictive factor for both treatment strategies [2, 3].

Genetically, CM is a heterogeneous malignancy [4–7]. Major oncogenic driver mutations in *BRAF* in 50–60% or *NRAS* in 15–30% highlight the important role of the mitogen-activated protein kinase (MAPK) pathway [8, 9]. However, patients with *NRAS* mutations are at special risk as this is associated with increased local recurrence and early metastatic spread [10, 11]. Besides, therapies for *NRAS* mutated melanoma are currently limited to immunotherapy or chemotherapy, as clinical trials targeting *NRAS* are just ongoing (*see clinicaltrials.gov: NCT04835805*). Besides, alterations in *NF1* or *MAP2K1* are found [12, 13]. Apart from that, there are manifold mutations that follow activations of the MAPK pathway as second hit, such as TERT promotor mutations or loss of tumor suppressors PTEN or p16^INK4A^, encoded by the *CDKN2A* gene, [4, 6, 14]. Furthermore, CM shows mutations associated with exposure to ultraviolet light (UV), so called UV signatures [4, 6]. Last, mutations of *TP53*, which is commonly found in tumor cells, or *EZH2*, as part of the chromatin-remodeling complex, and many infrequent mutations were described [4, 6].

So far, no mutations have been revealed that drive organ-specific, metastatic spread of CM [6, 15, 16]. Admittedly, mutations of both *NRAS* and *BRAF* are described to correlate with brain and liver metastasis [9]. But this cannot be used as reliable clinical predictor of hepatic metastasis since these mutations are very common. Selective hepatic metastasis of CM is associated with tumor-intrinsic expression of the homeobox protein MSX1 [11]. For lung metastasis of CM a CRISPRa screen provides deep genetic insights and links Fut7, Mgat5, Pcdh7, Olfr322, Olfr441 and Tmem116 to tumor progression [17]. Regarding tumor-intrinsic mechanisms regulating pulmonary metastasis of CM many different factors are described. As such, pulmonary colonization is enhanced by the activation of the melanoma cell adhesion molecule by S100A8/A9 and critically controlled by the histidine-rich glycoprotein in the plasma [18, 19]. Besides, expression of acetylglucosaminyltransferase is involved in lung metastasis, especially in extravasation of melanoma cells [20]. Moreover tumor-intrinsic expression of Apelin in CM promotes lung metastasis and outgrowth of metastases by blood and lymph vessel formation [21]. Furthermore, pulmonary colonization is affected by expression of angiopoetin-2 which protects CM cells from oxidative stress [22]. Last, tumor-intrinsic expression of CXCL5, a chemokine, regulates immunosuppressive neutrophiles and promotes lung metastasis of CM [23].

By in vivo selection techniques organotropic sublines may be generated which are an optimal tool to study drivers or mechanisms of organ-specific metastasis [24]. One of the most used CM cell lines, B16F10, was generated with this technique and was shown to more frequently and reliably generate lung colonies [25, 26]. Hepatic passaging of a B16 melanoma subline improves proclivity to hepatic metastasis by enhanced expression of integrin alpha2 and binding to collagen IV [27]. The role of integrins for liver metastasis is confirmed by a study that shows increased b4 integrin expression in a CRC subline after 10 passages via the liver [28]. Besides, in a fibrosarcoma model Amigo2 is upregulated and promotes adhesion to the hepatic vasculature [29]. All these studies share the same passaging route as the liver was colonized by spleen injections. In addition, serial passaging of melanoma cells may also simulate processes in long term survivors with stage IV melanoma [30]. Often several therapeutic approaches need to be chosen which results in repeated cycles of remissions and relapses and likely includes seeding of tumor cells from sites of metastasis.

B16 melanoma is widely used as experimental model of CM. However, underlying mutations and the response to immune checkpoint inhibition strongly differ from other cell lines and human patients [31, 32]. In contrast, WT31 melanoma is driven by a *NRAS* mutation resembling a cohort of patients with unmet clinical need, even colonizes the liver after intravenous (i.v.) injection and therefore better reflects the broader metastatic pattern of human patients [33, 34]. In our study we aimed at analyzing tumor-intrinsic mechanisms of hepatic melanoma metastasis. To this end, this selection process was performed after i.v. injection of WT31 melanoma cells to better model hematogenous spread of tumor cells.

## Methods

The methods described are established experimental techniques in our laboratory that in part also have been published in previous studies [34–36].

### Animals

For in vivo experiments female C57Bl/6 wildtype mice were purchased by Janvier. For metastasis experiments mice were age-matched and were used between 11 to 13 weeks of age. All animals were hosted in single ventilated cages (Sealsafe plus DGM™, Techniplast, Italy; Bedding H0234-20, Ssniff, Germany) in a 12 h/12 h day/night cycle under specific-pathogen-free conditions and fed ad libitum with a standard rodent diet (ssniff®R/M-H autoclavable, V1534-000, Ssniff, Germany).

### Cell lines

The murine melanoma cell line WT31 was derived from *Tyr∷NrasQ61K/°; INK4a*^*−/−*^ mice [33] and was a generous gift from O. Sansom (Beatson Institute for Cancer Research, Scotland). The WT31_P5IV subline was established by 5 cycles of hepatic passaging of WT31 after i.v. injection into the tail vein. Both the parental cell line WT31 and its subline WT31_P5IV were regularly tested mycoplasma-free by PCR. They were maintained in RPMI 1640 media (Thermo Fisher Scientific, Waltham, USA) with 10% (v/v) fetal calf serum (FCS) and 100 U/ml penicillin/streptomycin at 37 °C, 5% CO_2_. For all in vivo experiments the same passages of WT31 or WT31P5IV were used. After thawing of the cells they were not passaged more than three times and maximum culture time prior to in vivo experiments was one week.

### Hepatic passaging of WT31

To achieve vasodilatation of the veins, mice were put in a heating chamber at 37 °C for 10 min. Mice were anesthetized with isoflurane and 5 × 10^5^ WT31 melanoma cells were injected into the *V.caudalis lateralis*, typically referred to as tail vein. After 21 days the mice were sacrificed. Livers were removed, macroscopic metastases were dissected and meshed through 100 µm and 70 µm cell strainers. Isolated cells were resuspended in medium and cultured at 37 °C and 5% CO_2_. After at least 5 passages no remnants of the hepatic microenvironment were detected. Melanoma cells were expanded and were injected again into the tail vein*.* This cycle was repeated 5 times to generate the subline WT31_P5IV.

### In vivo adhesion and retention assay of WT31 and WT31_P5IV

WT31 and WT31_P5IV were detached at sub-confluency (50%), incubated at 1 × 10^6^ cells per ml with Dil-fluorescence dye (Invitrogen, Waltham, USA) for 20 min and washed 3 times with PBS. Injections into the *V.caudalis lateralis* were performed as described above. After 90 min mice were sacrificed, lungs and livers were dissected and put on a petri dish. Ex vivo imaging was performed by an IVIS200 charge-coupled device imaging system (Caliper Life Sciences, Waltham, USA) at an excitation wavelength of 535 nm. A red filter with an emission passband of 575 to 650 nm was used to detect DsRed emission. The quantification was performed with Living Image 4.1. (PerkinElmer, Waltham, USA).

### Liver dissection, cryopreservation and paraffin embedding

Mice were sacrificed by cervical dislocation. Livers were either fixed in 4% PFA at 4 °C for 24 h to 72 h, followed by paraffin embedding according to standard protocols or livers were embedded in OCT (Sakura Finetek Europe B.V. KvK, Alphen aan den Rijn, Netherlands).

### Immunohistochemistry and immunofluorescence

Acoording to standard protocols deparaffinization and rehydration of paraffin Sects. (3 µm) was performed. Antigen retrieval was carried out with epitope retrieval solution (Zytomed Systems, Berlin, Germany) at either pH 6 or pH 9 for 45 min at 95 °C. Cryosections (8 µm) were dried for 60 min at room temperature and were fixed with 4% PFA. First antibody was incubated at 4 °C over night, after three washing steps with PBS fluorophore-conjugated secondary antibody was applied for 1 h at room temperature. Sections were mounted with Dako fluorescent mounting medium (Dako, Agilent technologies, Santa Clara, USA).

For immunohistochemical staining tissue sections were blocked with Dako peroxidase solution (S2023, Agilent Technologies, Santa Clara, USA) for 10 min. Primary antibodies incubated over night at 4 °C followed by incubation with horseradish peroxidase (HRP)-conjugated secondary antibody at room temperature for 1 h. Dako AEC substrate chromogen (K3464, Agilent Technologies, Santa Clara, USA) was added for 30 min. After that tissue sections were counterstained with Mayer’s hemalum solution (1.09249.2500, Merck, Darmstadt, Germany) diluted 1:2 with ddH_2_O for 90 s. and mounted with Dako aqueous mounting medium (S3025, Agilent Technologies, Santa Clara, USA).

For hematoxylin & eosin (H&E), periodic acid-Schiff (PAS), Sirius red (SR) and Elastica-van Gieson (EvG) staining, formalin-fixed, paraffin-embedded samples were processed according to standard protocols provided by the manufacturer.

### Cell staining

5 × 10^4^ cells were seeded on cover slips in a 24 well plate. Cells were expanded for 24 h and fixed with 4% PFA for 5 min. Afterwards, cells were permeabilized with Triton X for 2 min and blocked with 5% normal donkey serum (NDS) in PBS for 1 h. Primary Antibodies were diluted in 1% NDS in PBS and incubated for 24 h at 4 °C. The next day cover slips were washed, fluorophore-conjugated secondary antibodies were also diluted in 1% NDS in PBS and cells were incubated for 1 h at RT with secondary antibodies. Last, cover slips were washed with PBS and mounted with DAKO fluorescent mounting medium on a slide for microscopy.

### Image acquisition and processing

Pictures of routine histology, immunofluorescence and cell staining were acquired by an Eclipse Ni-E motorized upright microscope (Nikon Instruments Europe BV, Amsterdam, Netherlands) using 10x, 20x or 40x CFI Plan Apochromat Lambda series objective lenses, an Intensilight Epifluorescence Illuminator, a DS-Ri2 high-definition color camera and a DS-Qi2 high-definition monochrome camera. The system was controlled by NIS-Elements AR 5.02 software (Nikon Instruments, Tokyo, Japan). During acquisition data were not compressed. Fluorescence images were acquired as z-stacks. Image processing included background reduction, deconvolution and extended depth of focus using NIS-Elements AR 5.02 and ImageJ software (NIH, USA). Quantification of immunofluorescence staining was performed by ImageJ software (NIH, USA). In detail, color thresholds were set in relation to the whole image (= fluorescent area).

### Antibodies

Primary antibodies: Rabbit anti-cleaved Caspase 3 (9661S, Cell Signaling Technology, Danvers, USA), rabbit anti-Ki-67 (ab16667, Abcam, Cambridge, UK), rat anti-CD31 (DM3614P, Dianova, Hamburg, Germany), goat anti-CD32b (AF1460, R&D Systems, Minneapolis, USA), goat anti-Lyve1 (AF2125, R&D Systems, Minneapolis, USA), rat anti-Endomucin (14–5851–82, Thermo Fisher Scientific, Waltham, USA), rabbit anti-TRP-2 (ab74073, Abcam, Cambridge, UK), rabbit anti-wide spectrum cytokeratin (ab9377, Abcam, Cambridge, UK), goat anti-alpha smooth muscle actin (ab21027, abcam, Cambridge, UK), rat anti-CD45 (550539, BD Biosciences, Franklin Lakes, USA), rat anti-CD68 (137002, BioLegend, San Diego, USA), rabbit anti-Melan A (NBP1-30151, Novus, Minneapolis, USA). For Western blotting: rabbit anti-Akt (9272S, Cell Signaling Technology, Danvers, USA), rabbit anti-phospho-Akt (9271S, Cell Signaling Technology, Danvers, USA). Secondary antibodies: Alexa-Fluor 488, Alexa-Fluor 647 and Cy3-conjugated secondary antibodies were purchased from Dianova (Hamburg, Germany). For Western Blotting a rabbit anti-IgG HRP conjugated antibody was used. (Merck, Darmstadt, Germany).

### RNA isolation and RNA sequencing

Total RNA from sub-confluent (50%), cultured melanoma cells was extracted with innuPREP RNA Mini Kit (845-KS-2080250, Analytik Jena, Jena, Germany), then treated with TURBO DNA-free Kit (AM1907, Invitrogen, Waltham, USA). Samples were prepared and RNA concentration and quality were measured by a NanoPhotometer NP80 (Implen, Munich, Germany) and a 2100 Bioanalyzer (Agilent Technologies, Santa Clara, USA). The library preparation and the sequencing were then performed by BGI (Hongkong, China). The raw and normalized gene expression profiling data have been deposited in NCBI's Gene Expression Omnibus and are accessible through GEO Series accession number GSE219236.

### RNA sequencing data analysis

Most of the procedure was done with R and Bioconductor using the NGS analysis package systempipeR [37]. The quality control of raw sequencing reads was performed using FastQC (Babraham Bioinformatics, Babraham, UK). Low-quality reads were removed using trim_galore (version 0.6.4). The resulting reads were aligned to mouse genome version GRCm38.p6 from GeneCode and counted using kallisto version 0.46.1 [38]. The count data was transformed to *log2*-counts per million (logCPM) using the voom-function from the limma package [39]. Differential expression analysis was performed using the limma package in R. A false positive rate of α = 0.05 with FDR correction was taken as the level of significance. Volcano plots and heatmaps were created using ggplot2 package (version 2.2.1) and the complex heatmap (version 2.0.0) [40]. Pathway analyses were made with fgsea package [41] and the enrichment browser package [42] in R using the pathway information from KEGG database (URL: https://www.genome.jp/kegg/pathway.html). The GSEA was made with R. Besides, a protein–protein interaction network was plotted by STRING (http://version10.string-db.org).

### Quantitative PCR

Reverse transcription was performed using Maxima Reverse Transcriptase (EP0752, Thermo Fisher Scientific, Waltham, USA) according to the manufacturer’s instructions. For quantitative PCR (qPCR) of cDNA innuMIX qPCR SyGreen Sensitive (845-AS-1310200, Analytik Jena, Jena, Germany) was used on a qTOWER 3 G touch thermal cycler (Analytik Jena, Jena, Germany). qPCR primers were designed with NCBIs PrimerBLAST (https://www.ncbi.nlm.nih.gov/tools/primer-blast/). Primer sequences are listed in Suppl. Table 10. Analysis of qPCR output files were performed in qPCRsoft 4.1 (Analytik Jena, Jena, Germany) and normalized expressions (ddCt algorithm) were calculated using the reference genes Gak and Srp72.

### Seahorse ATP-rate assay

At first, a XF-24 plate was coated with 3 µg/cm^2^ fibronectin (Sigma-Aldrich, St.Louis, USA) for 45 min at RT. WT31 and WT31_P5IV cells were seeded one day prior to the assay with a density of 5000 cells/well on a XF-24 plate. 1 h prior to the analysis the medium was replaced with Seahorse RPMI Medium (Agilent Technologies, Santa Clara, USA) supplemented with Seahorse XF Glucose (1.0 M solution), Seahorse XF Pyruvate (100 mM solution) and Seahorse XF L-Glutamine (200 mM solution) and the cells were incubated at 37 °C and 0% CO_2_ for 45 min. During the experiment Oligomycin was injected first (1.5 µM) followed by injection of Rotenone/Antimycin A (0.5 µM). The oxygen consumption rate (OCR), extracellular acidification rate (ECAR), mito ATP production rate and glyco ATP production rate were automatically calculated by the Seahorse XF-24 analyzer (Agilent Technologies, Santa Clara, USA).

A normalization to the total cell number was performed according to manufacturer´s instructions with CyQuant Cell Proliferation Assay (Thermo Fisher Scientific, Waltham, USA).

### Protein isolation

Cells were seeded at 4.5 × 10^6^ cells per T175 cell culture flask one day prior to the assay. Next, cells were harvested by a enzyme-free cell dissociation solution (Sigma-Aldrich, St.Louis, USA). Cells were pelleted at 300 g for 5 min and washed twice with PBS. Cells were lysed with 200 µl RIPA lysis buffer containing a EDTA-free protease inhibitor (Sigma-Aldrich, St.Louis, USA) and a PhosStop phosphatase inhibitor (Sigma-Aldrich, St.Louis, USA) for 30 min on ice. After centrifugation at 10,800 rpm for 5 min, the supernatant was transferred to new tubes and the protein concentration was measured by a colorimetric DC protein assay (Bio Rad, Hercules, USA).

### Western blot

Laemmli buffer (Bio Rad, Hercules, USA) was added to protein samples of WT31 and WT31_P5IV and incubated at 95 °C for 10 min. 50 µg of protein was loaded to 4–20% gradient polyacrylamide gels (Bio Rad, Hercules, USA) for electrophoresis. The transfer to PVDF membranes (Bio Rad, Hercules, USA) was performed by a Trans-Blot Turbo Transfer System (Bio Rad, Hercules, USA). Next, membranes were incubated with 5% skim milk in PBS for blocking, which was followed by an incubation with a primary antibody at 4 °C overnight. After three washing steps with PBS and 0,1% Tween 20, the membranes were incubated with a secondary antibody for 1 h at room temperature. The targeted protein was detected by chemiluminescence with a Chemilumenscence Imager (Intas Science Imaging, Göttingen, Germany). A quantification by densitometry was performed with ImageJ software (NIH, USA).

### Statistical analysis

All statistical analyses and graphical displays were performed with GraphPad Prism8 (Graph Pad, USA) and mean ± SD is presented. For statistical analysis, an unpaired, two-tailed *t*-test was applied if data met the criteria of normality. Otherwise, Mann U test was used. For grouped analysis, a Dunn’s multiple comparisons test was applied when data were not distributed normally. Differences between data sets with *P* < 0.05 were considered statistically significant.

## Results

### Serial hepatic passaging of WT31 melanoma generated a subline with decreased potential to lung metastasis and a trend towards increased liver metastasis

**Fig. 1:**
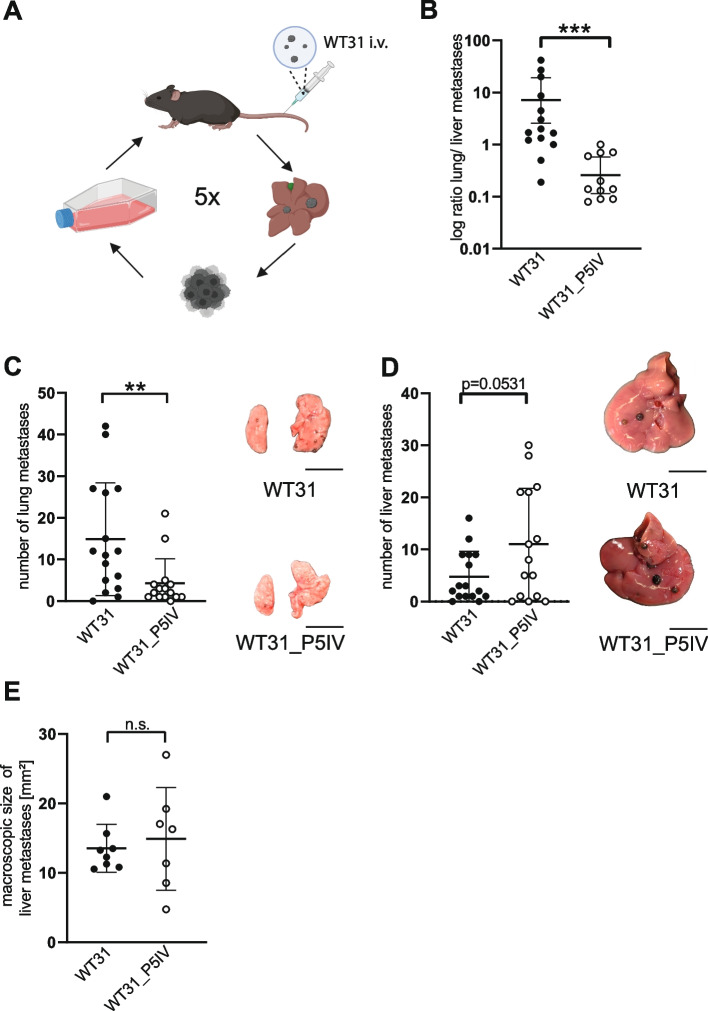
Repetitive passaging over the liver resulted in altered colonization of target organs after i.v. injection.** A** A scheme illustrates the serial injections and the hepatic passaging of WT31 melanoma or its sublines. WT31 was injected i.v.. After 21 days livers were excised and metastases were smashed through a cell strainer to get a single cell suspension. To expand these cells, they were taken back into cell culture. Last, sublines were again injected i.v.. This cycle was repeated five times generating the subline WT31_P5IV. **B** The ratio of lung to liver metastases of WT31 or WT31_P5IV was calculated and is shown (*P* = 0.0005, Mann–Whitney test) **C**, **D** 0.5 × 10^6^ WT31 and WT31_P5IV melanoma cells were injected i.v. and mice were sacrificed at day 21. **C** Macroscopic visible lung metastases were quantified. The numbers of macroscopic lung metastases of WT31 or WT31_P5IV melanoma are displayed (*P* = 0.0077, Mann–Whitney test). A pooled analysis of three different experiments is presented. Representative images of colonized lungs are shown. Scale bars = 1 cm. **D** Macroscopic visible liver metastases were counted. The number of macroscopic liver metastases of WT31 or WT31_P5IV are presented (*P* = 0.0531, t-test). Representative images of colonized livers are shown. Scale bars = 1 cm. **E** The size of liver metastases of WT31 or WT31_P5IV was measured and is presented in mm.^2^ (*P* = 0.6709, t-test)

Since patients with *NRAS*-mutated CM have a poor prognosis and limited treatment options, we aimed at deciphering tumor-intrinsic promotors of *NRAS*-mutated melanoma for metastasis to the lungs and the liver. WT31 melanoma was injected i.v. and repeatedly passaged over the liver five times to generate a hepatotropic subline, WT31_P5IV (Fig. 1A). This approach allows to study the metastatic pattern of WT31 and its sublines. Routinely, every passage was tested for purity by immunofluorescence staining (Suppl. Figure 1A). Moreover, mutant *NRAS*^*Q61K*/°^ was detected by PCR in parental WT31 and all its passaged subclones confirming cell authenticity (Suppl. Figure 1B).

To compare the metastatic behavior of the parental line WT31 and its subline WT31_P5IV the numbers of lung and liver metastases were quantified after i.v. injection. Metastatic behavior was profoundly altered as the ratio of lung to liver metastases was reversed in WT31_P5IV (*P* = 0.0005) (Fig. 1B). This was mediated by significantly decreased numbers of lung metastases (*P* = 0.0077) (Fig. 1C) and a trend towards increased numbers of liver metastases in WT31_P5IV in comparison to WT31 (*P* = 0.0531) (Fig. 1D). Among all sublines there was a trend to increased metastatic distribution at the third passage, while propensity for liver metastasis did develop at the fourth and fifth passage (Suppl. Figure 1C). In contrast to these numeric differences, the sizes of liver metastases were unaltered between both groups indicating similar tumor growth (*P* = 0.6709) (Fig. 1E).

### Lung metastases of WT31_P5IV were less proliferative, while morphology and necrosis were unaltered

**Fig. 2:**
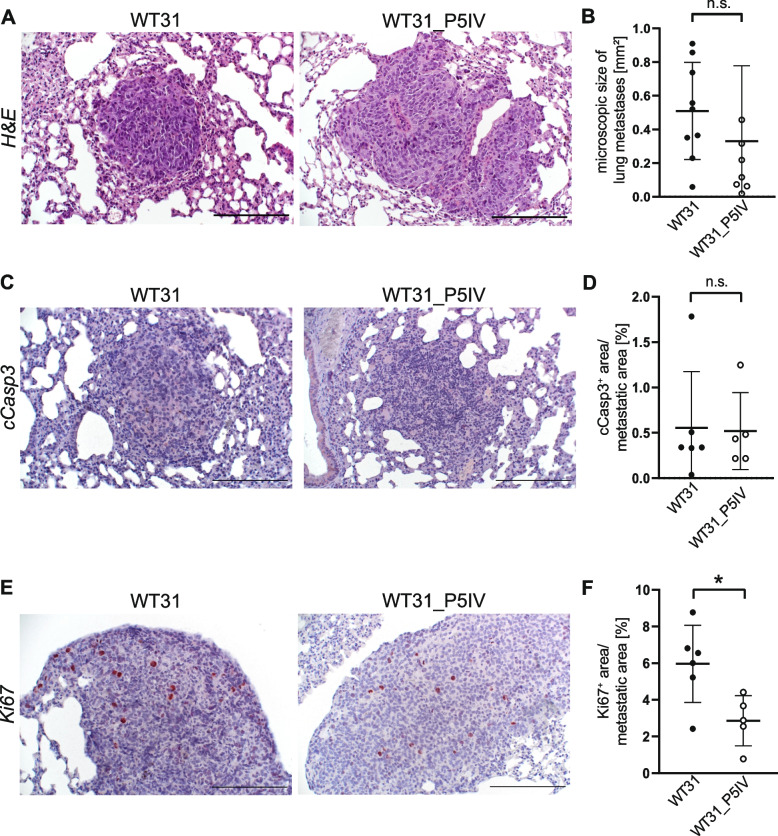
Histologic analysis of lung metastases of WT31 and WT31_P5IV. **A** H&E staining of lung metastases of WT31 and WT31_P5IV. Scale bars = 200 µm **B** The size of lung metastases was measured and the area of macroscopic lung metastases is presented (*P* = 0.3512). **C** Representative images of immunohistochemical staining for cCasp3 of lung metastases of WT31 and WT31_P5IV are shown. Scale bars = 200 µm. **D** Quantification of the area of cCasp3^+^ cells in relation to the total metastatic area of lung metastases (*P* > 0.9999, t-test, *n* = 6 for WT31 and *n* = 5 for WT31_P5IV). **E** Representative images of immunohistochemical staining for Ki67 in lung metastases of WT31 and WT31_P5IV are shown. Scale bars = 200 µm. **F** Quantification of the area of Ki67^+^ cells in relation to the total metastatic area of lung metastases (*P* = 0.0171, t-test, *n* = 6 for WT31 and *n* = 5 for WT31_P5IV)

To further characterize lung metastases of WT31_P5IV in comparison to its parental line WT31 detailed microscopic analyses were performed. H&E staining of lung metastases of WT31 and WT31_P5IV showed no overt differences in morphology (Fig. 2A). Besides, the microscopic sizes of the metastases were similar (Fig. 2B). Apoptosis and proliferation of melanoma lung metastases were studied by immunohistochemical staining for cleaved caspase-3 (cCasp3) and Ki67 to study if these factors in part mediate the decrease of pulmonary metastasis in WT31_P5IV. cCasp3 signals in lung metastases were similar in WT31_P5IV and WT31 (Fig. 2C, D). But proliferation as measured by Ki67 was decreased in lung metastases of WT31_P5IV indicating disturbed adaptation to the microenvironment of the lung (*P* = 0.0171) (Fig. 2E, F).

### Liver metastases of WT31_P5IV and WT31 showed similar morphology, vascularization, proliferation and necrosis

**Fig. 3:**
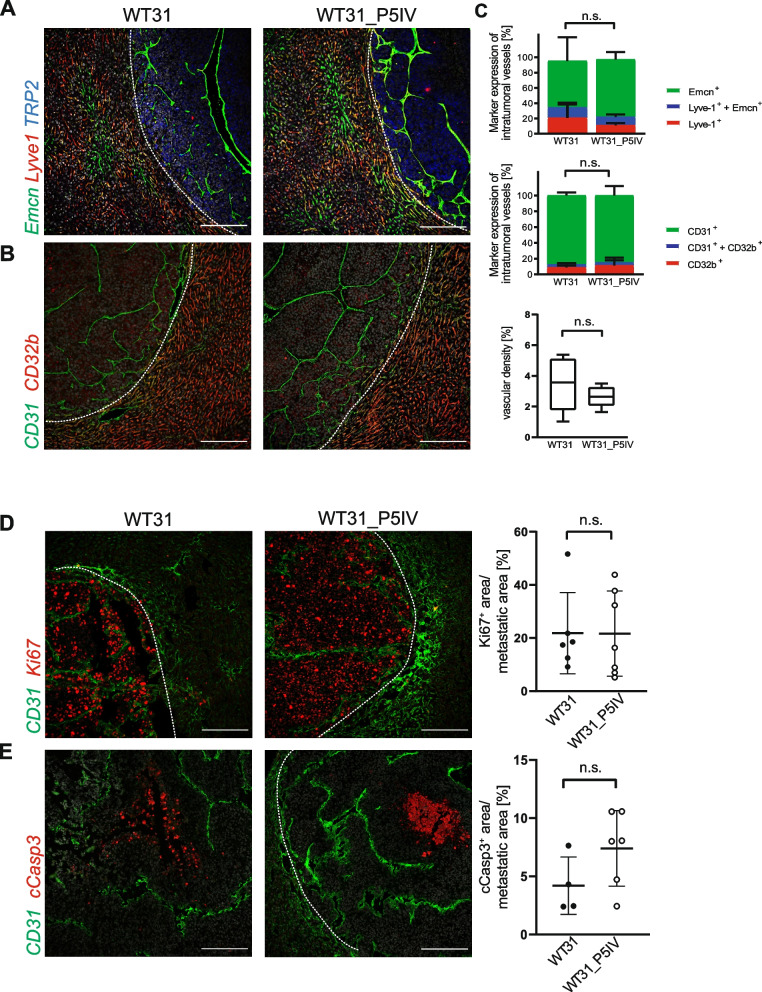
Analysis of the tumoral vasculature, proliferation and necrosis of liver metastases of WT31 and WT31_P5IV. **A** Images of immunofluorescence staining for Emcn (green), Lyve-1 (red) and TRP-2 (blue) of liver metastases of WT31 and WT31_P5IV. Representative images are shown. Scale bars = 200 µm, *n* = 4. White dotted lines present the border of hepatic metastasis of WT31 and WT31_P5IV to the adjacent liver tissue. **B** Images of immunofluorescence staining for CD31 (green) and CD32b (red) of liver metastasis of WT31 and WT31_P5IV. Representative images are presented. Scale bars = 200 µm, *n* = 5. White dotted lines present the border of hepatic metastasis of WT31 and WT31_P5IV to the adjacent liver tissue. **C** Intratumoral endothelial cells were analyzed for Emcn or Lyve-1 marker expression. The corresponding marker expression was set in relation to the total intratumoral vessel area (100%). The proportion of Lyve^+^, Lyve^+^  + Emcn^+^ as well as Emcn^+^ area is displayed (Emcn: *P* > 0.9999; Lyve-1 and Emcn: *P* = 0.7000; Lyve-1: *P* > 0.9999; Mann–Whitney-tests). Besides, the expression of CD31 and CD32b of intratumoral vessels was analyzed and set in relation to the total intratumoral vessel area (100%). The proportion of CD31^+^, CD31^+^  + CD32b^+^ as well as CD32b^+^ area is displayed. (CD31: *P* = 0.6325; CD31 and CD32b: *P* = 0.7118; CD32b: *P* = 0.6492; t-tests). Last, the vascular density of the hepatic melanoma metastases was determined. The CD31.^+^ area in relation to the total tumor area is shown (*P* = 0.3776, t-test) **D** Images of immunofluorescence staining for CD31 and Ki67 of liver metastases of WT31 and WT31_P5IV. Representative images are presented. Scale bars = 200 µm, *n* = 6. White dotted lines present the border of the hepatic metastasis of WT31 and WT31_P5IV to the adjacent liver tissue (*P* = 0.6282, Mann–Whitney test). **E** Images of immunofluorescence staining for CD31 and cCasp3 of liver metastases of WT31 and WT31_P5IV. Representative images are shown. Scale bars = 200 µm, *n* = 4. White dotted lines present the border of the hepatic metastasis of WT31 and WT31_P5IV to the adjacent liver tissue (*P* = 0.1163, t-test)

Liver metastases of both melanoma sublines were studied in detail to analyze whether hepatic passaging affected the tumor-intrinsic adaptation of WT31_P5IV to the hepatic microenvironment. Histologic analyses by H&E, Sirius red, Elastica van Gieson’s (EvG) or periodic acid-Schiff (PAS) staining revealed no differences (Suppl. Figure 2). Moreover, both subtypes of metastases showed a pushing type histopathological growth pattern. Next, the vascularization of liver metastases of WT31_P5IV and WT31 were analyzed by immunofluorescence staining for Endomucin and Lyve-1 as well as CD31 and CD32b (Fig. 3A, B). An expression of continuous endothelial cell markers and reduced expression of liver sinusoidal endothelial cell markers was seen in both hepatic metastases indicating a predominantly capillarized phenotype of intratumoral vessels (Fig. 3C). This was confirmed by immunofluorescences for Collagen IV and CD31 showing deposits of Collagen IV alongside CD31^+^ endothelial cells (Suppl. Figure 3A). Quantification of the staining showed no differences between both melanoma sublines and demonstrated that the vascular density was not changed between metastases of WT31_P5IV and WT31 (Fig. 3C). Last, proliferation and apoptosis were assessed by staining for Ki67 and cCasp3 showing no differences between WT31 and WT31_P5IV (Fig. 3D, E). Therefore, we hypothesized that serial passaging of WT31 melanoma over the liver might rather have influenced tumor-intrinsic functions that determine movement and retention along the vascular passaging route than adaptations of tumor growth and expansion in the hepatic microenvironment.

### RNA sequencing of WT31_P5IV and parental WT31 demonstrated differential regulation of cell adhesion pathways

**Fig. 4:**
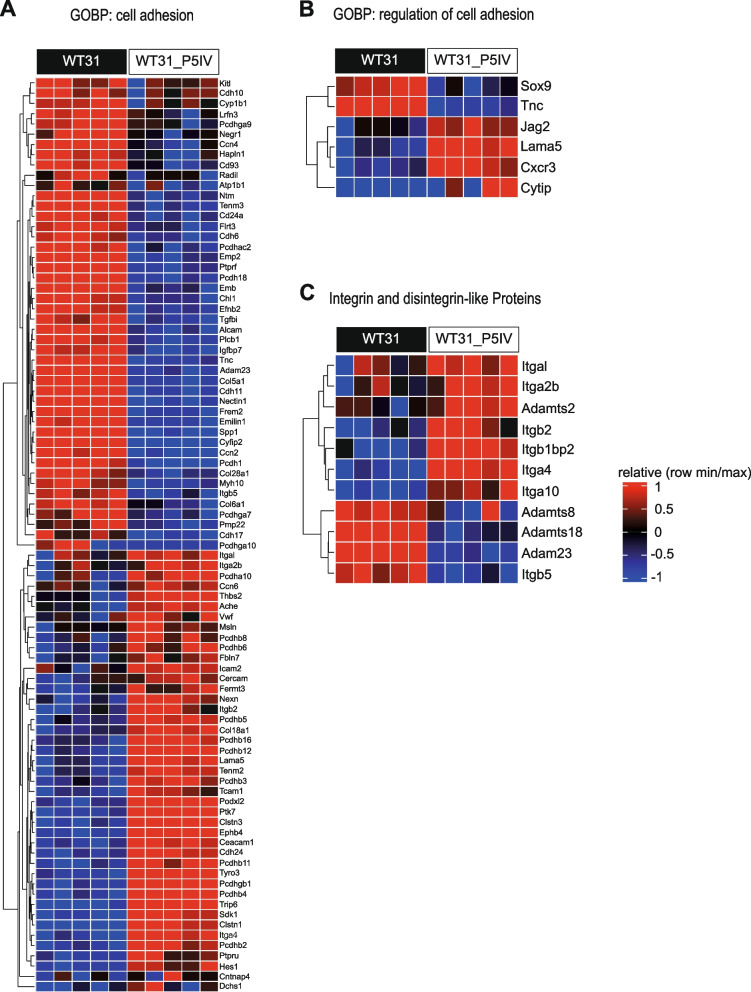
RNA sequencing of WT31 or WT31_P5IV. RNA sequencing of cultured, sub-confluent WT31 and WT31_P5IV melanoma cells was performed. **A** A heatmap of the GOBP cell adhesion gene set (GO:0,007,155) for WT31 and WT31_P5IV melanoma is presented. The red colored bars present upregulated genes, whereas blue colored bars show downregulation. Genes symbols are written on the right side. Genes were filtered by an adjusted *P* value < 0.05 and logFC ≥ 1 or ≤ -1. Genes are also listed in Suppl. Table 2. **B** A heatmap of the GOBP regulation of cell adhesion gene set (GO:0,030,155) of WT31 and WT31_P5IV melanoma is shown. The red colored bars present upregulated genes, whereas blue colored bars show downregulation. Genes symbols are written on the right side. Genes were filtered by an adjusted *P* value < 0.05 and logFC ≥ 1 or ≤ -1. Genes are also listed in Suppl. Table 3. **C** A heatmap of integrin and disintegrin-like proteins of WT31 and WT31_P5IV melanoma is shown. The red colored bars present upregulated genes, whereas blue colored bars show downregulation. Genes symbols are written on the right side. Genes were filtered by an adjusted *P* value < 0.05 and logFC ≥ 1 or ≤ -1. Genes are also listed in Suppl. Table 4

To further elucidate why WT31_P5IV showed an altered pattern of metastatic colonization we performed RNA sequencing (RNA-seq). Unfiltered comparative gene expression analysis and over-representation analysis (ORA) of gene ontology biological processes (GOBP) showed regulation of mostly metabolic processes (Suppl. Figure 4A). In the Hallmark gene sets genes involved in oxidative phosphorylation, glycolysis, Myc targets, mitotic spindle, DNA repair, PI3K/AKT/mTor signaling or Mtorc1 signaling were regulated (Suppl. Figure 4B). Filtering of these gene sets (logFC ≥ 1 or ≤ -1; adjusted *P* value < 0.05) demonstrated that glycolysis and oxidative phosphorylation seemed to be affected in WT31_P5IV (Suppl. Figure 4C-F, Suppl. Tables 2–9). However, the mito ATP production rate and the glyco ATP production rate were unaltered between WT31 and WT31_P5IV as assessed by a Seahorse assay (Suppl. Figure 5). Some of the significantly up- or downregulated genes were confirmed by qRT-PCRs (Suppl. Figure 6A-C). For instance, a significant downregulation of *Igfbp3* was detected (Suppl. Figure 6A). As IGFBP3 negatively interacts with AKT signaling [43], the expression of both phosphorylated AKT (pAKT) and total AKT (AKT) was analyzed by western blotting (Suppl. Figure 7A, B). The ratio of pAKT to AKT was significantly increased in WT31_P5IV confirming activation of this pathway (Suppl. Figure 7C). Interestingly, applying the same filtering criteria to the gene set of GOBP cell adhesion showed the highest number of regulated genes compared to other HALLMARK gene sets (Fig. 4A). Furthermore, genes classified as regulators of cell adhesion, integrins or disintegrin-like proteins were differentially regulated in WT31_P5IV in comparison to WT31 and were also confirmed by qRT-PCRs (Fig. 4B, C; Suppl. Figure 6C; Suppl. Table 2–4). A strong interaction of these genes was demonstrated by a string analysis of the top 100 most regulated genes of GOBP cell adhesion (Suppl. Figure 8A). Decreased adhesion of circulating melanoma cells to the pulmonary vasculature might indeed explain the inverted ratio from lung to liver metastases since tumor cells that do not adhere in the vascular bed of the first metastatic site are flushed into the next, secondary vascular beds.

### Tumor cell adhesion and retention of WT31_P5IV in the pulmonary vascular bed was reduced as compared to WT31

**Fig. 5:**
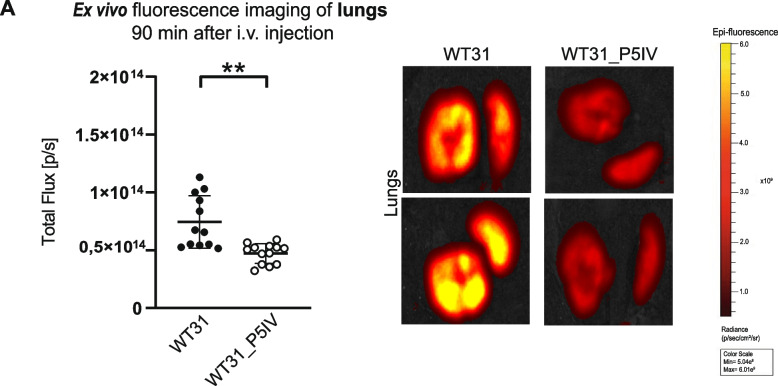
Initial tumor cell retention to the pulmonary vasculature was studied ex vivo after i.v. injection. **A** Ex vivo fluorescence imaging of lungs 90 min after i.v. injection of DIL-labelled WT31 or WT31_P5IV. Quantification of adherent WT31 or WT31_P5IV melanoma cells. Lungs were set as regions of interest and fluorescence signal was quantified respectively (*P* = 0.0016, t-test). *N* = 4/group, the experiment was repeated three times. Scale: min = 5.04e^8^ p/sec/cm^2^/sr; max = 6.01e^9^ p/sec/cm^2^/sr

Initial tumor cell adhesion and retention were investigated 90 min after intravenous injection of DIL-labelled WT31 or WT31_P5IV by ex vivo fluorescence imaging. Reduced retention of WT31_P5IV to the vascular bed of the lungs was confirmed, as significantly fewer fluorescent melanoma cells in the lungs were found when WT31_P5IV were injected i.v. in relation to WT31 (*P* = 0.0016) (Fig. 5A). In the liver no difference in melanoma cell adhesion or retention was detected (Suppl. Figure 9A). However, auto-fluorescence of the liver might in part conceal differences. Besides, the cell numbers of this assay were optimized to study initial melanoma cell adhesion and retention to the lung vasculature. Probably at later time points an increasing amount of accumulating melanoma cells in the hepatic vasculature might be found as these first disseminate to various organs after the pulmonary passage.

Altogether, our study shows that tumor-intrinsic features such as tumor cell adhesion decisively regulate the vascular route tumor cells take and hereby control the metastatic patterns of melanoma.

## Discussion

Cutaneous melanoma is a prime example of organ-specific metastasis as it commonly spreads to the skin, lymph nodes, lung, liver and brain. Organotropic metastasis is also observed in breast cancer preferential seeding to lungs, bone, liver and brain, prostate cancer that almost solely metastasizes to the bones or uveal melanoma (UM) which selectively seeds to the liver. These phenomena led Stephan Paget to postulate his concept of “seed & soil” defining that circulating tumor cells depend on suitable microenvironments of their target organs to sufficiently grow out as metastasis [44]. In contrast, James Ewing hypothesized that rather circulatory effects affect organotropic metastasis [45]. Our study demonstrates that hepatic passaging of *NRAS*-mutated melanoma, WT31, shifts its typical metastatic propensity by regulation of tumor cell adhesion at the primary vascular site and adaptation to the metastatic site.

By serial hepatic passaging of WT31 melanoma cells after i.v. injection a subline, WT31_P5IV, was generated that showed an inverted ratio of lung to liver metastases as compared to parental WT31. According to Ewing this could be mediated by altered tumor cell adhesion and retention in the primary, pulmonary vascular niche resulting in increasing numbers of circulating tumor cells that might enter secondary vascular beds such as the liver. Indeed, ex vivo fluorescence imaging confirmed that fewer WT31_P5IV melanoma cells adhered to the lungs 90 min after i.v. injection as compared to WT31 indicating that initial tumor cell adhesion and retention to the lungs was reduced in WT31_P5IV. RNA-seq of both WT31 and its subline WT31_P5IV revealed differential regulation of genes involved in cell adhesion. When looking at the top regulated genes several have already been implied in tumor cell adhesion. Loss of Collagen XXIII reduced pulmonary colonization of lung carcinoma cell lines by impaired cell adhesion [46]. Besides, downregulation of *IGFBP7* (Insulin Like Growth Factor Binding Protein 7) was detected in CRC liver metastases and shown to regulate epithelial‑mesenchymal transition (EMT) [47]. Downregulation of *Cdh11* (Cadherin-11) might also result in reduced tumor cell adhesion as Cadherin-11 was identified as regulator of cell adhesion and metastasis in many cancers [48–50]. On the other hand, upregulation of *THBS2* (Thrombospondin2) was detected in liver metastases of UM or CRC and might serve as a prognostic biomarker for both tumor entities [51, 52]. Moreover, integrins are described to mediate organ-specific melanoma metastasis [53]. As such, *Itga4* which was upregulated in WT31_P5IV is associated with melanoma lymph node metastasis [53]. Besides, upregulation of *Itga2b* in WT31_P5IV our data is in line with previous studies of Yoshimura et al. that passaged B16 melanoma after spleen injection [27]. Data from the Cancer Genome Atlas indicate that increased levels of *ITGB2* (Integrin β2) are a poor prognostic factor for UM [54]. In part, our study contrasts with previous in vivo selections of CRC or fibrosarcoma which share upregulation of different adhesion molecules after hepatic passaging [28, 29]. However, these studies used spleen injection to colonize the liver. Therefore, we conclude that the vascular route of in vivo tumor passaging strongly influences the resultant characteristics of the subline and may also provide insight into selection processes that occur in human patients during disease progression. We hypothesize that further passaging of WT31_P5IV via the lungs or generation of a novel subline with passaging via the liver as first vascular bed might considerably affect the selection process and probably significantly increase the number of hepatic metastases. The influence of the interaction of endothelial cells with melanoma cells is highlighted by a recent study. Upon interaction with vascular niches the mesenchymal phenotype of melanoma cells, which is crucially determined by *Prrx1*, is suppressed and tumor growth is promoted in a hierarchical fashion [55].

Furthermore, in vivo passaging influenced adaptation to the microenvironment of metastatic sites, the liver and the lungs, in the sense of Paget. As such, the proliferation of lung metastases of WT31_P5IV was significantly reduced in relation to parental WT31, whereas liver metastases of both cell lines showed no differences. However, parental WT31 are already well adapted to the hepatic microenvironment as they efficiently colonize the liver even after i.v. injections and form large and highly vascularized liver metastases [34]. Therefore, induction of strong differences of liver metastases themselves by in vivo passaging might not be expected. But regulation of tumor promoting genes was identified by RNA-seq of WT31_P5IV as compared to parental WT31. One of the most-upregulated genes was *Capg* (gelsolin-like actin-capping protein) which was identified as poor prognostic factor for various cancers, especially liver metastasis of CRC [56, 57]. Moreover, *Apoe* (Apolipoprotein E) was upregulated which is a hub gene for liver metastasis of CRC and a negative predictive factor [58, 59]. On the contrary, strongly downregulated genes included *Igfbp3* (insulin like growth factor binding protein 3) that led to downstream activation of AKT. This is in line with previous studies in CM demonstrating that downregulation of *IGFBP3* promotes melanoma cell survival, proliferation and invasion via phosphorylation of AKT [43]. Furthermore, *Sox2* (SRY-Box Transcription Factor 2) was one of the top five most-downregulated genes. Among other functions, Sox2 is involved in regulation of oxidative phosphorylation [60]. This might explain over-representation of genes with relevance for oxidative phosphorylation in the Hallmarks pathway analysis. However, by Seahorse assays no significant differences in oxidative phosphorylation or glycolysis between WT31 and its passaged subline were detected. Last, strongly downregulated genes included *Pcdh18*, a tumor suppressor in CRC, and *Nectin* which might drive melanoma cell proliferation upon loss of these genes [61, 62]. Besides, ADAMTS18, which is also described as potential tumor-suppressor in melanoma, was also strongly downregulated [63].

Altogether, we demonstrate that tumor-intrinsic features of *NRAS*-mutated melanoma cells are decisively controlled by the vascular route tumor cells take during organ colonization. Selection of tumor cells by repeated in vivo hepatic passaging, which includes travelling of tumor cells through the circulation, led to downregulated tumor cell adhesion and retention to the first vascular bed and inverted the ratio of lung to liver metastases. Similar processes seem to occur in patients that survive stage IV melanoma. Therefore, this needs to be considered when aiming to prevent metastatic spread of melanoma.

## Conclusions

Serial hepatic passaging of *NRAS* mutated melanoma strongly affected its metastatic pattern. Specifically, initial tumor cell adhesion to the pulmonary vasculature was decreased allowing the melanoma cells to easier colonize the liver. This is important for the understanding of the molecular processes underlying metastasis. Repeated tumor cell seeding and colonization may also influence metastatic patterns in human patients with advanced, recurrent melanoma which received multiple treatments.

## Supplementary Information

**Additional file 1:** **Supplementary Figures.** **Suppl. Fig. 1.** Verification of cell purity and identity by immunofluorescence staining and PCR. A. Melanoma cells from liver metastases were excised and expanded in cell culture. To verify cell purity every subline was stained for Melan A, CD68, αSMA, Desmin, CD31, CD45 or pan cytokeratin before i.v. injection. Representative immunofluorescence images of passaged WT31 after isolation from liver metastases and expansion in cell culture are shown. Melanoma cells were stained with MelanA (green). Besides CD68 (hepatic macrophages), CD31 (endothelial cells), alphaSMA (fibroblasts), CD45 (hematopoietic cells), Desmin (myogenic differentiated cells, myofibroblasts) or pan-cytokeratin (hepatocytes) were stained in red respectively, Scale bars = 200µm B. PCR of human NRAS of WT31 and sublines P1, P2, P3, P4 and P5 (WT31_P5IV). H2O and B16F10 luc2 melanoma serve as negative controls. Product length: 499 bp. C. The number of organs colonized by WT31 melanoma and its sublines WT31_P1, WT31_P2, WT31_P3, WT31_P4 and WT31_P5 is shown. The number of lung metastases for each subline is presented (*P* = 0.0193 for WT31_P1IV vs. WT31_P2IV, *P* = 0.0107 for WT31_P2IV vs. WT31_P5IV). The number of liver metastases are displayed (*P* = 0.0028 for WT31_P2IV vs. WT31_P4IV, *P* = 0.0189 for WT31_P2IV vs. WT31_P5IV) A Dunn’s test was performed respectively. Number of animals analyzed = 5 (WT31_P1IV), 7 (WT31_P2IV), 6 (WT31_P3IV), 6 (WT31_P4IV), 4 (WT31_P5IV). **Suppl. Fig. 2.** H&E, Sirius Red, EvG and PAS staining of liver metastases of WT31 and WT31_P5IV. Images of H&E (A), Sirius Red (B), EvG (C) and PAS (D) staining of hepatic metastases of WT31 and WT31_P5IV melanoma. Scale bars = 200µm, *n*=5, black dotted lines show border of metastases to normal liver tissue. **Suppl. Fig. 3.** Analysis of extracellular matrix deposits by immunofluorescence staining for Collagen IV. A. Images of immunofluorescence staining for CD31 and Collagen IV of liver metastases of WT31 and WT31_P5IV. Representative images are displayed. Scale bars = 200µm, n=5. White dotted lines present the border of hepatic metastasis of WT31 and WT31_P5IV to the adjacent liver. The Collagen IV^+^ area was quantified in relation to the tumoral area (*P* = 0.4858, t-test). **Suppl. Fig. 4.** Over-representation analysis (ORA) of gene ontology biological processes (GOBP) or HALLMARK pathways. RNA sequencing of cultured, sub-confluent WT31 and WT31_P5IV melanoma cells was performed and the gene sets were compared by an over-representation analysis of gene ontology biological processes (GOBP) (A) or HALLMARK pathways (B). Dot plots display both the number of regulated genes by count and gene ratio and the adjusted *P* value of corresponding pathways. C. Heatmaps of HALLMARK oxidative phosphorylation and glycolysis of WT31 and WT31_P5IV melanoma are shown. The red colored bars present upregulated genes, whereas blue colored bars show downregulation. Genes symbols are written on the right side. Genes were filtered by an adjusted *P* value < 0.05 and logFC ≥ 1 or ≤ -1. Genes are also listed in Suppl. Table 5 and 6. D. A heatmap of HALLMARK mitotic spindle of WT31 and WT31_P5IV melanoma is shown. The red colored bars present upregulated genes, whereas blue colored bars show downregulation. Genes symbols are written on the right side. Genes were filtered by an adjusted *P* value < 0.05 and logFC ≥ 1 or ≤ -1. Genes are also listed in Suppl. Table 7. E. A Heatmap of HALLMARK DNA repair of WT31 and WT31_P5IV melanoma is shown. The red colored bars present upregulated genes, whereas blue colored bars show downregulation. Genes symbols are written on the right side. Genes were filtered by an adjusted *P* value < 0.05 and logFC ≥ 1 or ≤ -1. Genes are also listed in Suppl. Table 8. F. A Heatmap of HALLMARK PI3K/AKT/mTOR-signaling of WT31 and WT31_P5IV melanoma is shown. The red colored bars present upregulated genes, whereas blue colored bars show downregulation. Genes symbols are written on the right side. Genes were filtered by an adjusted *P* value < 0.05 and logFC ≥ 1 or ≤ -1. Genes are also listed in Suppl. Table 9. **Suppl. Fig. 5.** Mito ATP production rate (*P* = 0.4145) and glyco ATP production rate (*P* = 0.1908) of WT31 and WT31_P5IV measured by Seahorse XF analysis. A. Mito ATP production rate and glyco ATP production rate in WT31 and WT31_P5IV were measured by a Seahorse XF analysis 24 h after seeding by consecutive injections of oligomycin (1µM) and antimycin A / rotenone (0.5 µM). The experiment was repeated three times. A pooled analysis of the mean values for either the mito ATP production rate and the glycoATP production rate is presented for WT31 and WT31_P5IV. Besides, representative graphs of OCR (pmol/min/cells) and ECAR (mpH/min/cells) are shown. Red line = WT31_P5IV; blue line = WT31. **Suppl. Fig. 6.** Quantitative real-time PCRs (qRT-PCR) of selective genes in parental WT31 in comparison to WT31_P5IV. A.Selected genes of Top 25 strongly up- or downregulated genes in WT31_P5IV are shown in comparison to parental WT31. Upregulated genes: *Capg* (*P *< 0.0001, t-test), *Gm773* (*P *= 0.0079, Mann-Whitney test), *Gpm6a* (*P *= 0.0244, t-test), *Pcdhgb8* (*P *< 0.0001, t-test); downregulated gene: *Igfbp3* (*P *< 0.0001, t-test). The normalized expression values were calculated using the Pfaffl method. Two reference genes (*Gak* and *Srp72*) were used for normalization. Suppl. Table 1 lists TOP 25 up- and downregulated genes in WT31 and WT31_P5IV whereas Suppl. Table 10 lists the Primers which were used for qRT‑PCRs. B.Selected up- and downregulated genes of HALLMARK mitotic spindle in WT31_P5IV are shown in comparison to parental WT31. Upregulated genes: *Notch2* (*P *< 0.0001, t-test), *Sun2* (*P *= 0.0001, t-test); downregulated genes: *Arhgef3* (*P *< 0.0001, t-test), *Sorbs2 *(*P* < 0.0001, t‑test). The normalized expression values were calculated using the Pfaffl method. Two reference genes (*Gak* and *Srp72*) were used for normalization. Suppl. Table 10 lists the primers which were used for qRT‑PCRs. C. Selected up- and downregulated genes of GOBP cell adhesion as well as selected integrin and disintegrin-like proteins in WT31_P5IV are shown in comparison to parental WT31. Upregulated genes: *Itga2b* (*P *= 0.0079, Mann-Whitney test), *Itga4* (*P *< 0.0001, t-test); downregulated genes: *Adam23* (*P *< 0.0001, t-test), *Itgb5 *(*P* < 0.0001, t-test), *Adamts18*(*P* = 0.0002, t-test). The normalized expression values were calculated using the Pfaffl method. Two reference genes (*Gak* and *Srp72*) were used for normalization. Suppl. Table 10 lists the Primers which were used for qRT‑PCRs. **Suppl. Fig. 7. **Western blot for total AKT (AKT) and phosphorylated AKT (pAKT) in WT31 and WT31_P5IV. A. A western blot of total AKT and GAPDH in WT31 and WT31_P5IV is shown. The original marker bands are shown at the left side. The experiment was repeated two times. Besides, a quantification of AKT in correlation to GAPDH by densitometry was performed with ImageJ and is presented on the right side of the western blots. B. A western blot of phosphorylated AKT (pAKT) and GAPDH in WT31 and WT31_P5IV is shown. The original marker bands are shown at the left side. The experiment was repeated two times. Besides, a quantification of pAKT in correlation to GAPDH by densitometry was performed by ImageJ and is presented on the right side of the western blots. C. The ratio of normalized pAKT/normalized AKT of each individual sample was calculated and is shown for WT31 in comparison to WT31_P5IV (*P* = 0,0291, t-test). Pooled data of two individually performed experiments are presented. **Suppl. Fig. 8.** Protein-protein interaction network by STRING analysis of GOBP: cell adhesion. A. A STRING analysis of GOBP: celladhesion displays possible protein-protein interactions. Up- and downregulated genes with an adjusted *P *< 0.05 were included into the analysis. Light blue line = known interactions from curated databases, violet line = known interactions experimentally determined, green line = textmining, black line = co-expression, pale violet line = protein homology, blue line = predicted interactions in gene co-occurrence. **Suppl. Fig. 9.** Initial tumor cell adhesion and retention to the liver vasculature was studied *ex vivo* for WT31 and WT31_P5IV after i.v. injection. A. *Ex vivo* fluorescence imaging of livers 90 min after i.v. injection of DIL labelled WT31 or WT31_P5IV. Quantification of adherent WT31 or WT31_P5IV melanoma cells. Livers were set as regions of interest and fluorescence signal was quantified respectively (*P *= 0.5031, Mann-Whitney test). *N* = 4/group, the experiment was repeated three times. Scale: min = 5.04e^8^ p/sec/cm^2^/sr; max = 6.01e^9^/sec/cm^2^/sr.

**Additional file 2: Supplementary Tables.** **Table 1.** A. Top twenty-five significantly upregulated genes of RNA Sequencing of WT31 and WT31_P5IV ordered by logFC. B. Top twenty five significantly downregulated genes of RNA Sequencing of WT31 and WT31_P5IV ordered by logFC. **Table 2.** A.Significantly upregulated genes of GO:0007155 (cell adhesion) of WT31 and WT31_P5IV filtered by logFC ≥ 1. B. Significantly downregulated genes of GO:0007155 (cell adhesion) of WT31 and WT31_P5IV filtered by logFC ≤ -1. **Table 3.** A.Significantly upregulated genes of GO:0030155 (regulation of cell adhesion) of WT31 and WT31_P5IV filtered by logFC ≥ 1. B. Significantly downregulated genes of GO:0030155 (regulation of cell adhesion) of WT31 and WT31_P5IV filtered by logFC ≤ -1. **Table 4.** A. Significantly upregulated genes of Integrin and disintegrin-like proteins of WT31 and WT31_P5IV filtered by logFC ≥ 1. B. Significantly downregulated genes of Integrin and disintegrin-like proteins of WT31 and WT31_P5IV filtered by logFC ≤ -1. **Table 5.** A. Significantly upregulated genes of HALLMARK oxidative phosphorylation of WT31 and WT31_P5IV filtered by logFC ≥ 1. B. Significantly downregulated genes of HALLMARK oxidative phosphorylation of WT31 and WT31_P5IV filtered by logFC ≤ -1. **Table 6.** A. Significantly upregulated genes of HALLMARK glycolysis of WT31 and WT31_P5IV filtered by logFC ≥ 1. B. Significantly downregulated genes of HALLMARK glycolysis of WT31 and WT31_P5IV filtered by logFC ≤ -1. **Table 7.** A. Significantly upregulated genes of HALLMARK mitotic spindle of WT31 and WT31_P5IV filtered by logFC ≥ 1. B. Significantly downregulated genes of HALLMARK mitotic spindle of WT31 and WT31_P5IV filtered by logFC ≤ -1. **Table 8.** A. Significantly upregulated genes of HALLMARK DNA Repair of WT31 and WT31_P5IV filtered by logFC ≥ 1. B. Significantly downregulated genes of HALLMARK DNA-Repair of WT31 and WT31_P5IV filtered by logFC ≤ -1. **Table 9.** A. Significantly upregulated genes of HALLMARK PI3K/AKT/MTOR-signaling of WT31 and WT31_P5IV filtered by logFC ≥ 1. B. Significantly downregulated genes of HALLMARK PI3K/AKT/MTOR-signaling of WT31 and WT31_P5IV filtered by logFC ≤ -1. **Table 10.** A.Primers used for qRT-PCR.  

## Data Availability

The raw and normalized gene expression profiling data have been deposited in NCBI's Gene Expression Omnibus and are accessible through GEO Series accession number GSE219236.

## Abbreviations

AKT: Total AKT
cCasp3: Cleaved caspase-3
CM: Cutaneous melanoma
CRC: Colorectal carcinoma
ECAR: Extracellular acidification rate
ICI: Immune checkpoint inhibition
LSEC: Liver sinusoidal endothelial cells
OCR: Oxygen consumption rate
pAKT: Phosphorylated AKT
RNA-seq: RNA sequencing
UM: Uveal melanoma
TT: Targeted therapy
