# Supplementary material for: Hepatic passaging of NRAS-mutant melanoma influences adhesive properties and metastatic pattern
Source: BMC Cancer. 2023 May 13;23:436. doi: 10.1186/s12885-023-10912-4 (PMC10182637; doi:10.1186/s12885-023-10912-4)

Suppl. Figure 1

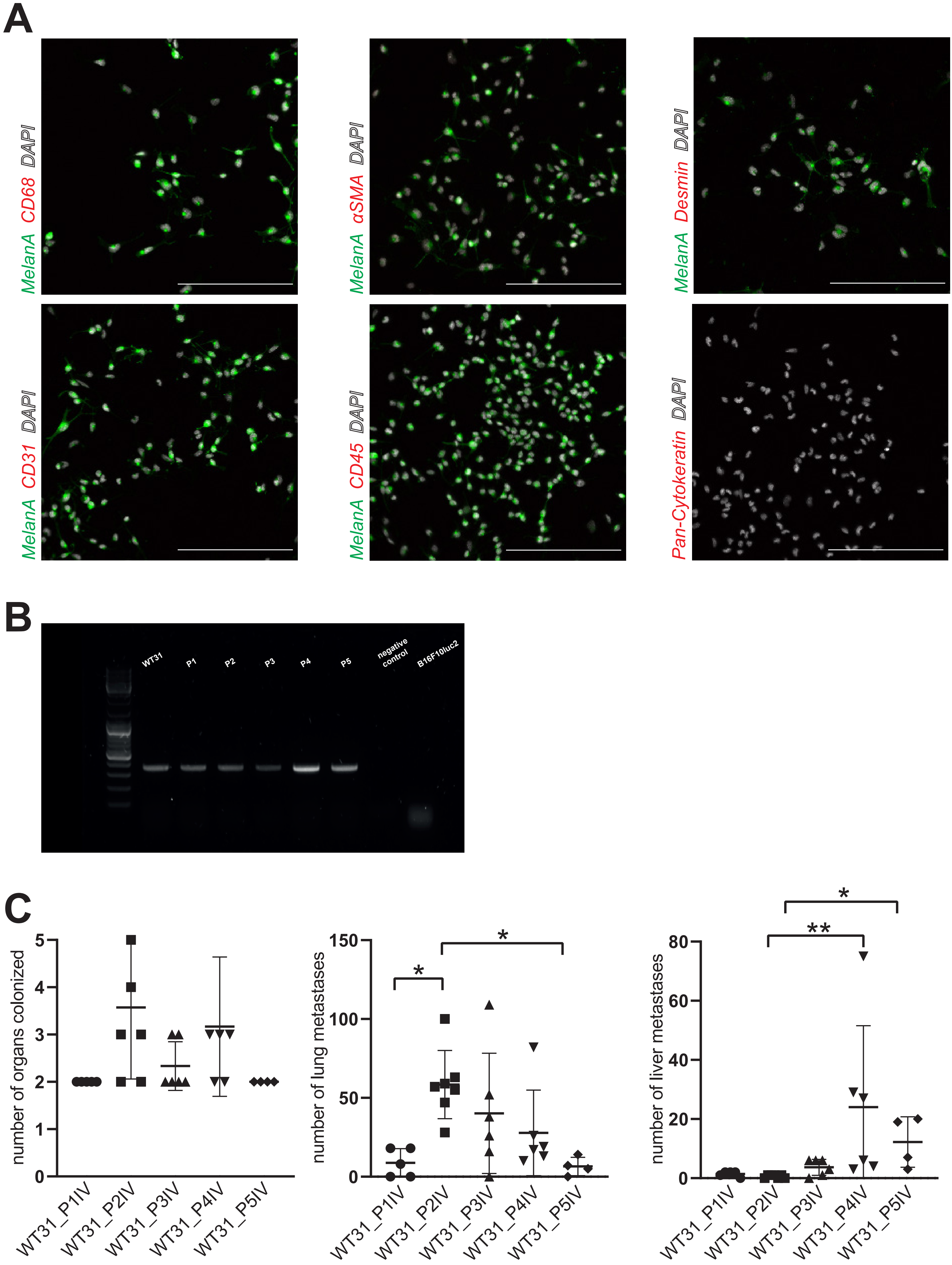

Suppl. Figure 2

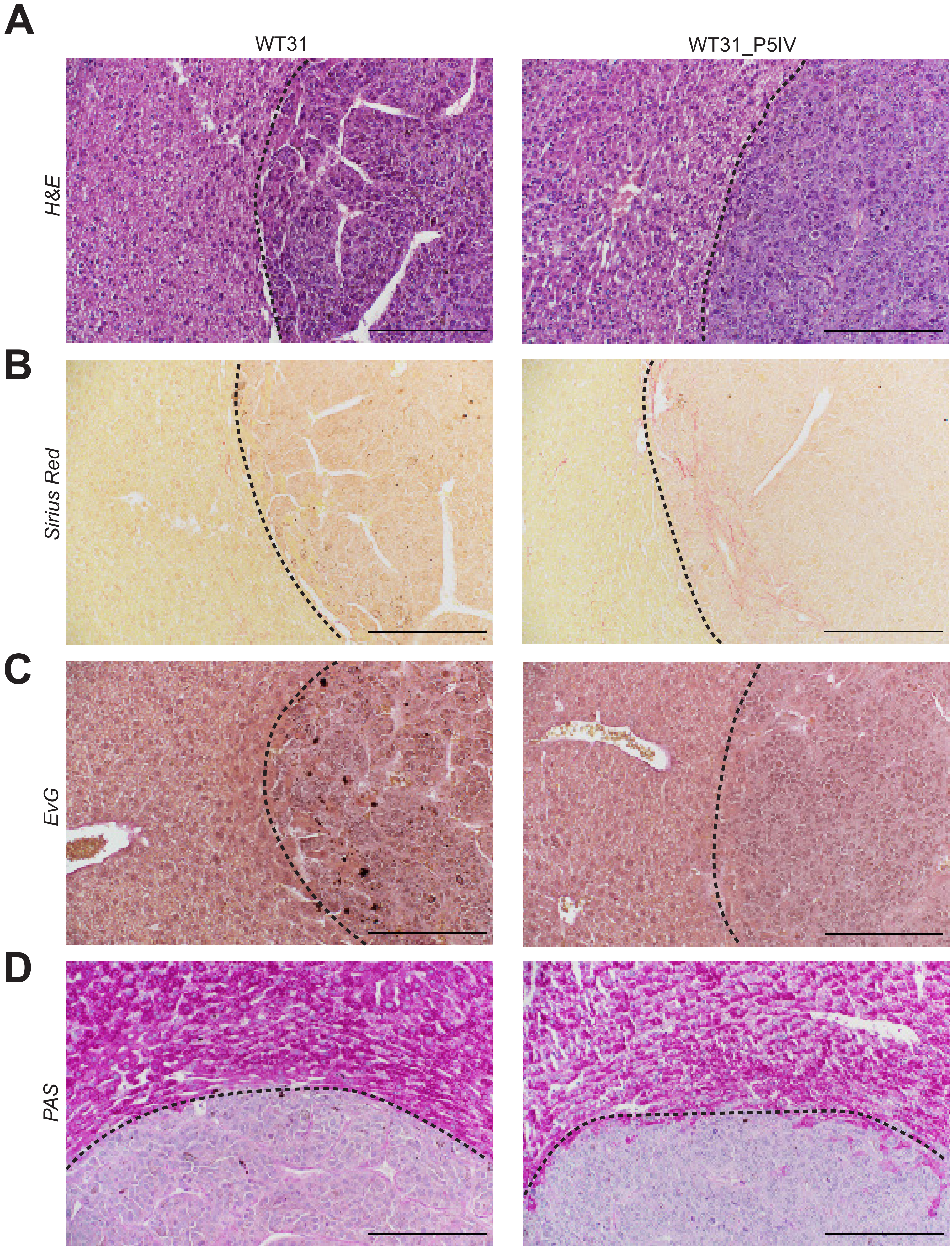

Suppl. Figure 3

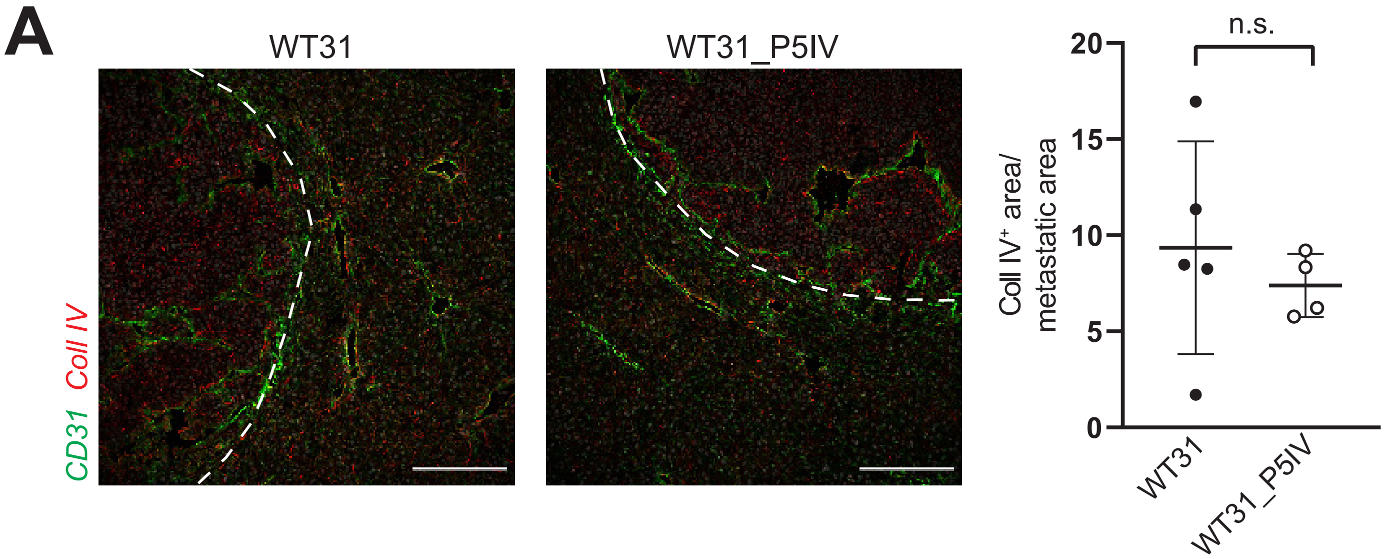

Suppl. Figure 4

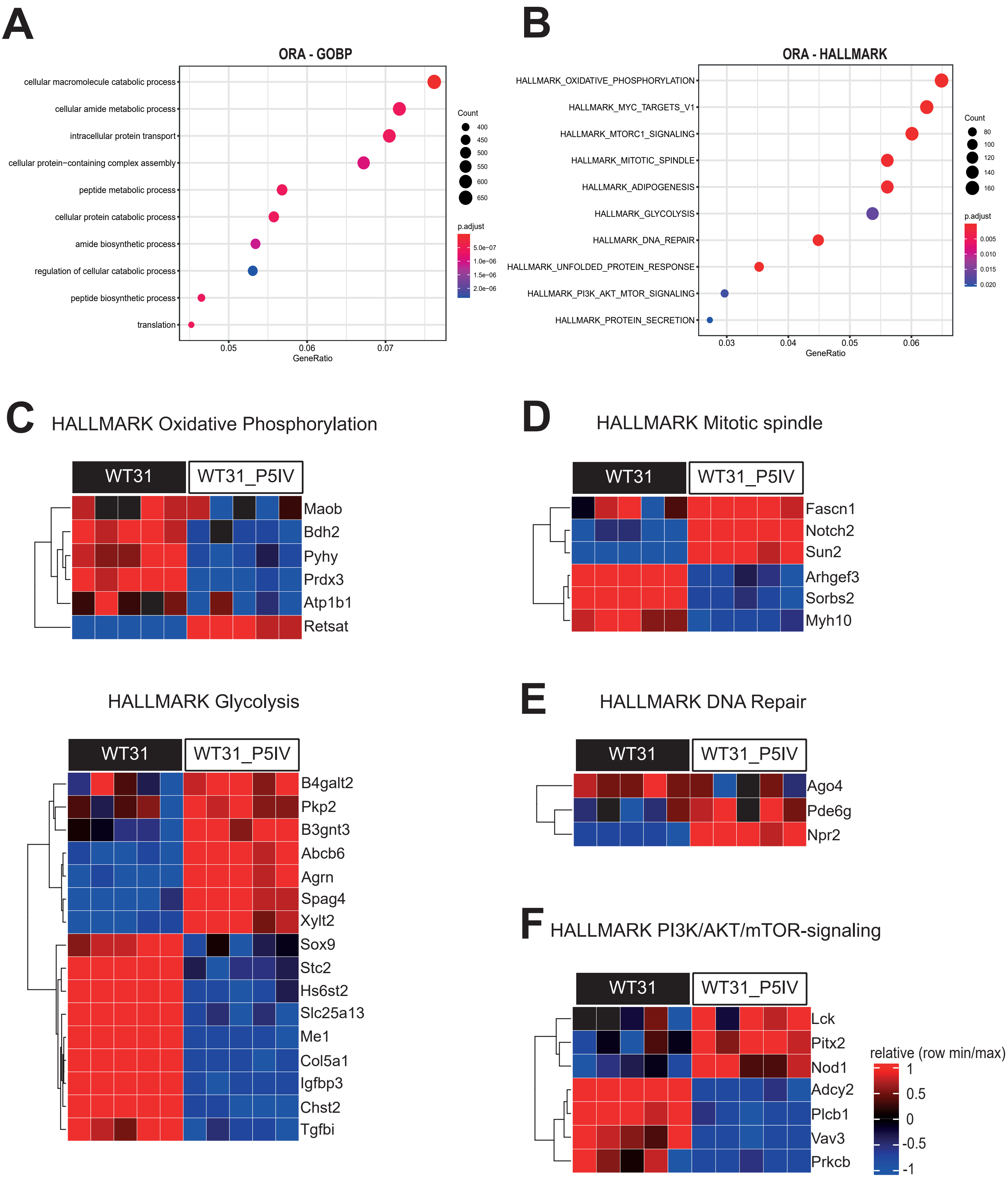

Suppl. Figure 5

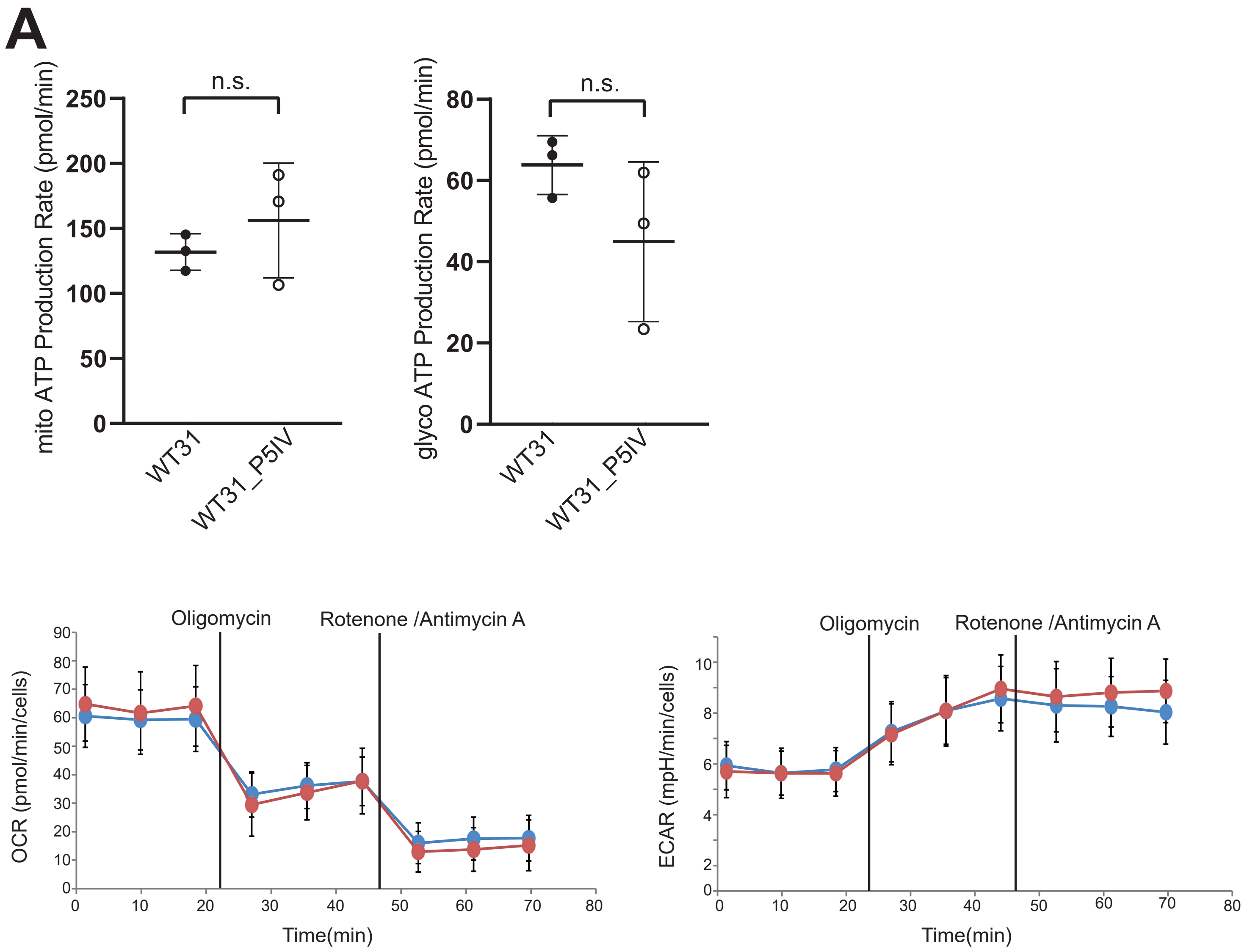

Suppl. Figure 6

A Selected genes of Top 25 up- and downregulated genes

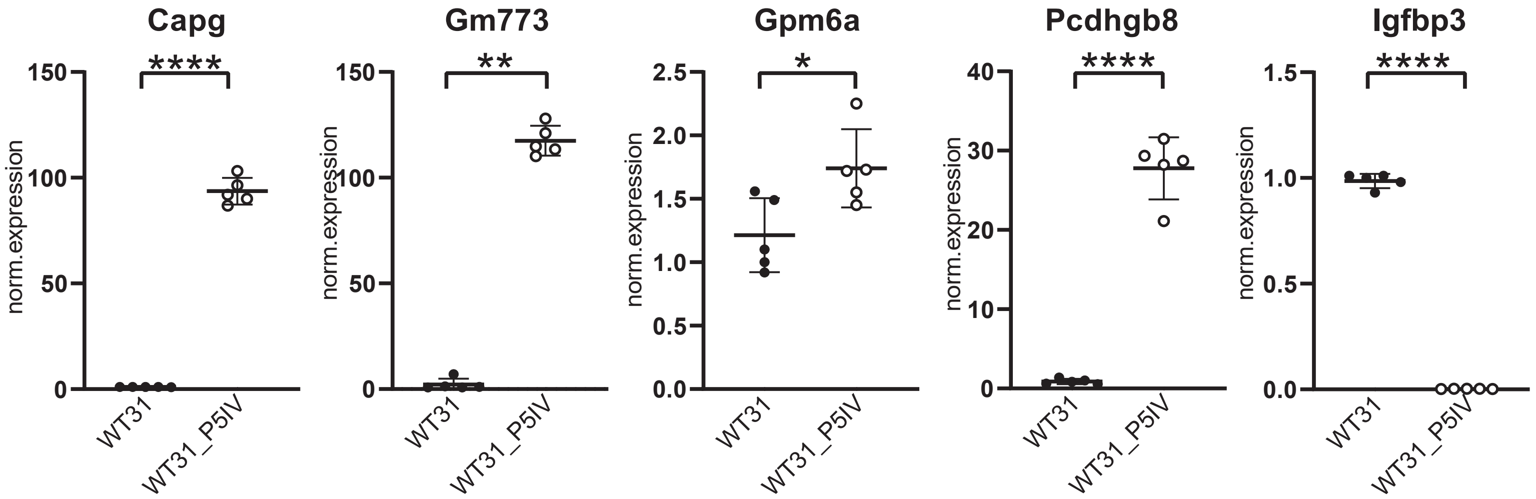

B Selected genes of HALLMARK mitotic spindle

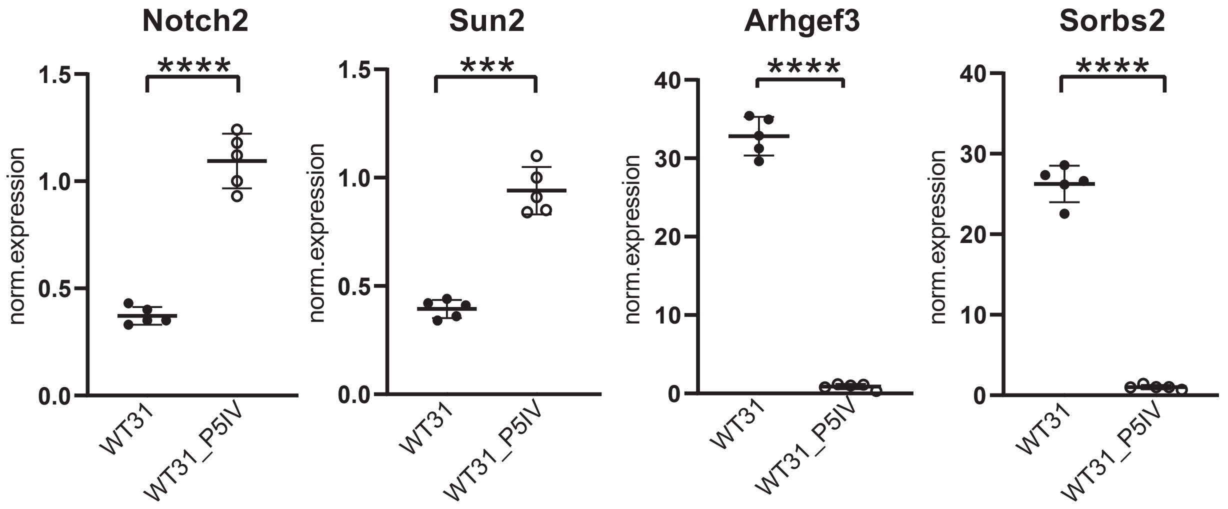

C Selected genes of GOBP cell adhesion and genes of integrins and disintegrin-like proteins

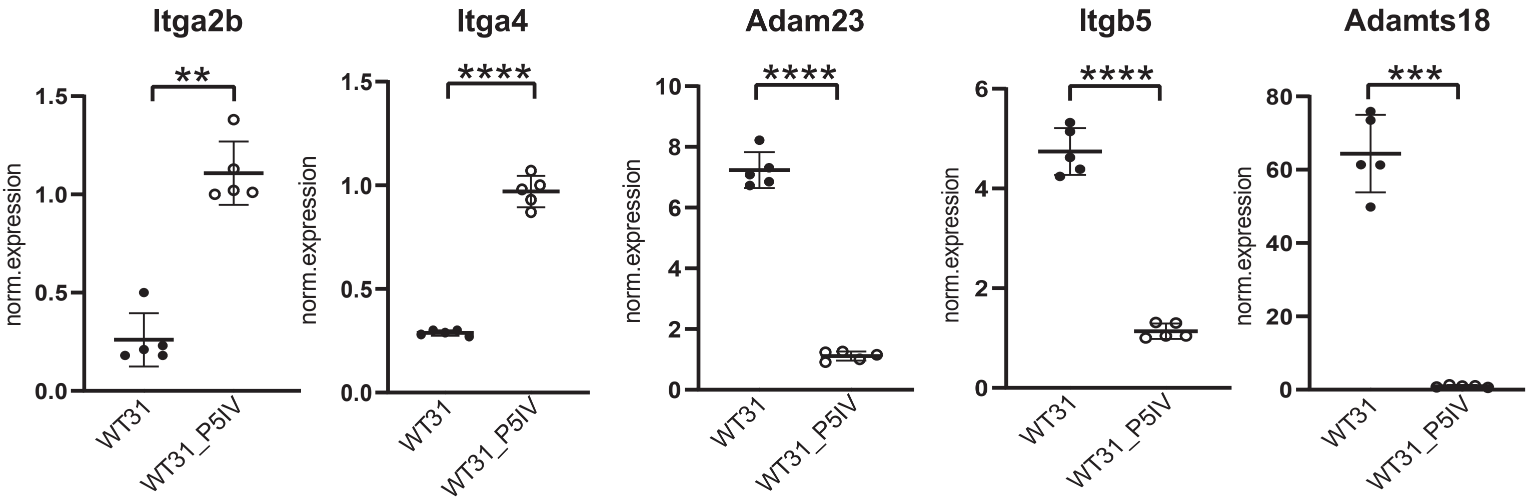

Suppl. Figure 7

A Western blot - AKT

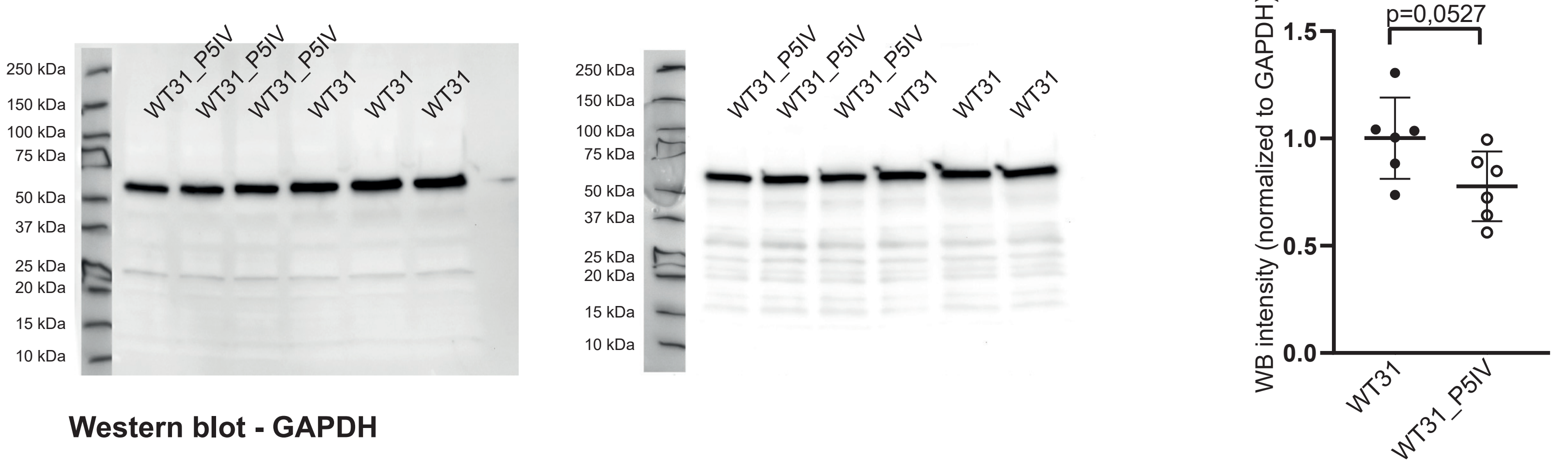

Western blot - GAPDH

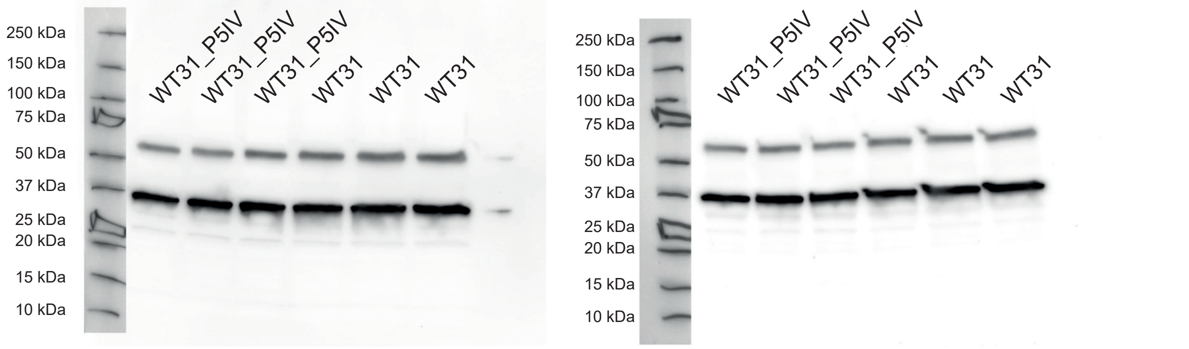

B Western blot - pAKT

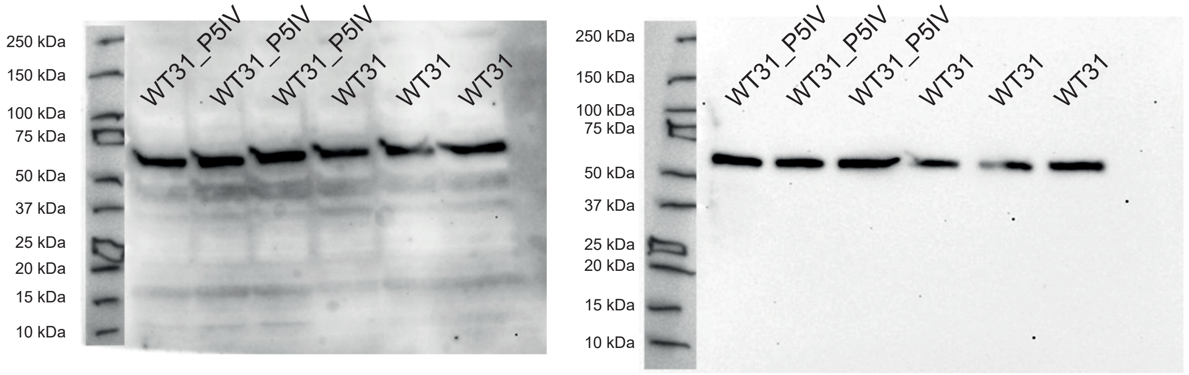

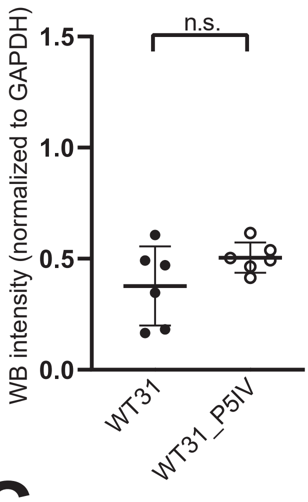

Western blot - GAPDH

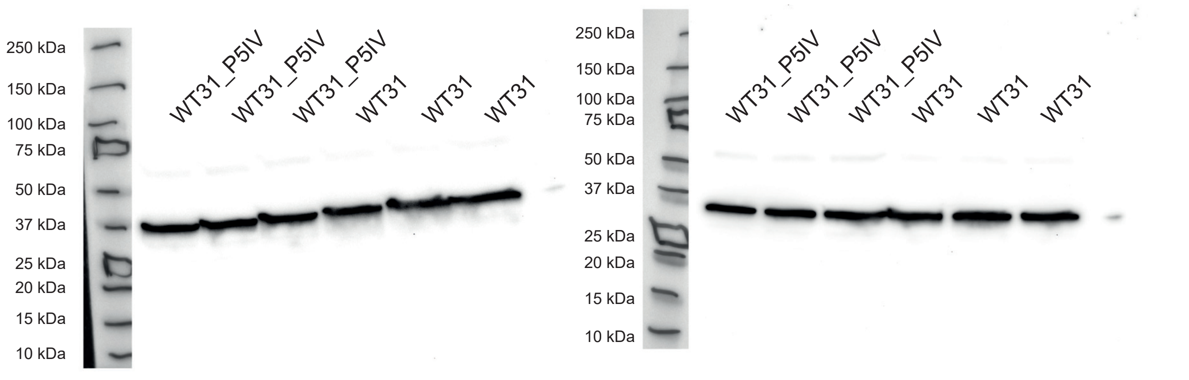

C

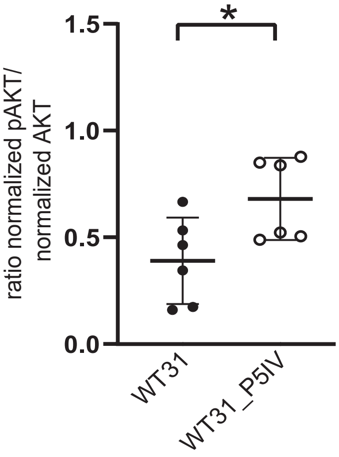

Suppl. Figure 8

A

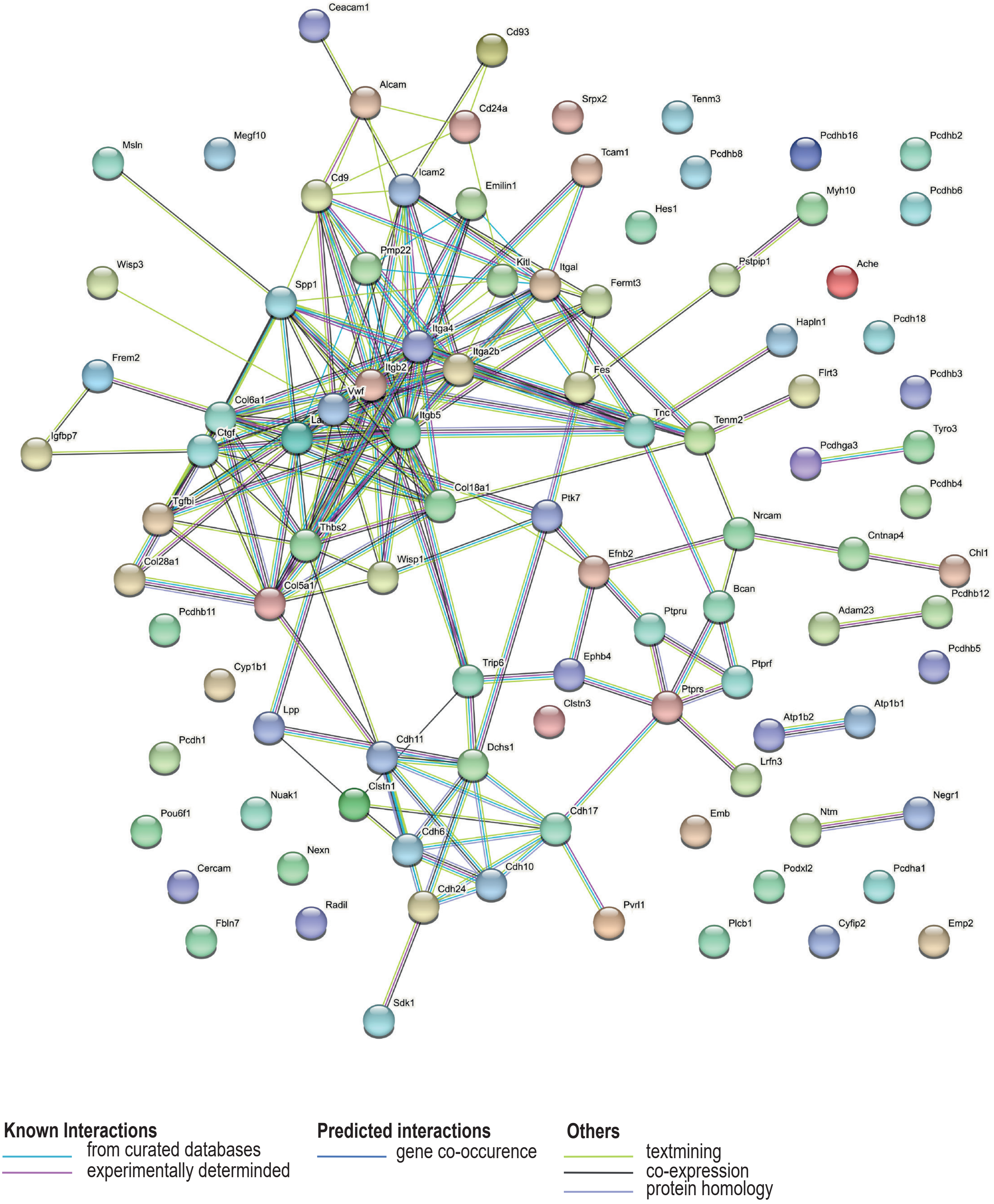

Suppl. Figure 9

A

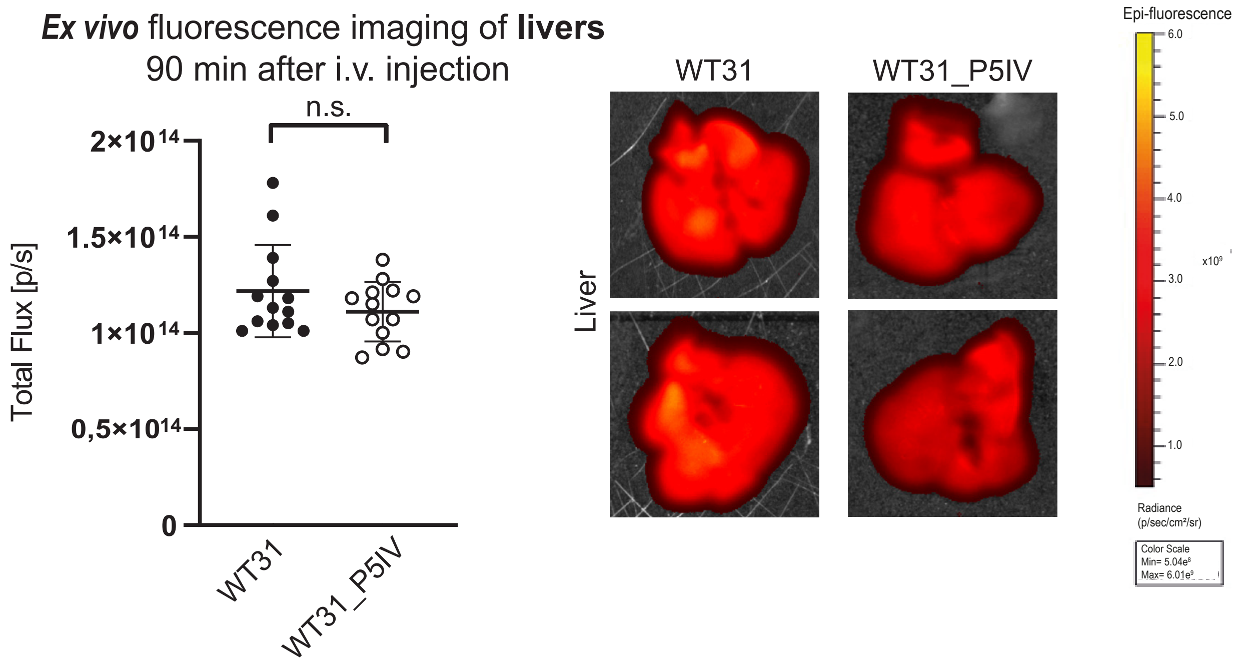

Supplement: Supplementary file 1 — Additional file 1: Supplementary Figures. Suppl. Fig. 1. Verification of cell purity and identity by immunofluorescence staining and PCR. A. Melanoma cells from liver metastases were excised and expanded in cell culture. To verify cell purity every subline was stained for Melan A, CD68, αSMA, Desmin, CD31, CD45 or pan cytokeratin before i.v. injection. Representative immunofluorescence images of passaged WT31 after isolation from liver metastases and expansion in cell culture are shown. Melanoma cells were stained with MelanA (green). Besides CD68 (hepatic macrophages), CD31 (endothelial cells), alphaSMA (fibroblasts), CD45 (hematopoietic cells), Desmin (myogenic differentiated cells, myofibroblasts) or pan-cytokeratin (hepatocytes) were stained in red respectively, Scale bars = 200µm B. PCR of human NRAS of WT31 and sublines P1, P2, P3, P4 and P5 (WT31_P5IV). H2O and B16F10 luc2 melanoma serve as negative controls. Product length: 499 bp. C. The number of organs colonized by WT31 melanoma and its sublines WT31_P1, WT31_P2, WT31_P3, WT31_P4 and WT31_P5 is shown. The number of lung metastases for each subline is presented (P = 0.0193 for WT31_P1IV vs. WT31_P2IV, P = 0.0107 for WT31_P2IV vs. WT31_P5IV). The number of liver metastases are displayed (P = 0.0028 for WT31_P2IV vs. WT31_P4IV, P = 0.0189 for WT31_P2IV vs. WT31_P5IV) A Dunn’s test was performed respectively. Number of animals analyzed = 5 (WT31_P1IV), 7 (WT31_P2IV), 6 (WT31_P3IV), 6 (WT31_P4IV), 4 (WT31_P5IV). Suppl. Fig. 2. H&E, Sirius Red, EvG and PAS staining of liver metastases of WT31 and WT31_P5IV. Images of H&E (A), Sirius Red (B), EvG (C) and PAS (D) staining of hepatic metastases of WT31 and WT31_P5IV melanoma. Scale bars = 200µm, n=5, black dotted lines show border of metastases to normal liver tissue. Suppl. Fig. 3. Analysis of extracellular matrix deposits by immunofluorescence staining for Collagen IV. A. Images of immunofluorescence staining for CD31 and Collagen IV of liver metastases of WT31 [file 12885_2023_10912_MOESM1_ESM.pdf]
