# Supplementary material for: Hepatic passaging of NRAS-mutant melanoma influences adhesive properties and metastatic pattern
Source: BMC Cancer. 2023 May 13;23:436. doi: 10.1186/s12885-023-10912-4 (PMC10182637; doi:10.1186/s12885-023-10912-4)
Supplement: Supplementary file 2 — Additional file 2: Supplementary Tables. Table 1. A. Top twenty-five significantly upregulated genes of RNA Sequencing of WT31 and WT31_P5IV ordered by logFC. B. Top twenty five significantly downregulated genes of RNA Sequencing of WT31 and WT31_P5IV ordered by logFC. Table 2. A.Significantly upregulated genes of GO:0007155 (cell adhesion) of WT31 and WT31_P5IV filtered by logFC ≥ 1. B. Significantly downregulated genes of GO:0007155 (cell adhesion) of WT31 and WT31_P5IV filtered by logFC ≤ -1. Table 3. A.Significantly upregulated genes of GO:0030155 (regulation of cell adhesion) of WT31 and WT31_P5IV filtered by logFC ≥ 1. B. Significantly downregulated genes of GO:0030155 (regulation of cell adhesion) of WT31 and WT31_P5IV filtered by logFC ≤ -1. Table 4. A. Significantly upregulated genes of Integrin and disintegrin-like proteins of WT31 and WT31_P5IV filtered by logFC ≥ 1. B. Significantly downregulated genes of Integrin and disintegrin-like proteins of WT31 and WT31_P5IV filtered by logFC ≤ -1. Table 5. A. Significantly upregulated genes of HALLMARK oxidative phosphorylation of WT31 and WT31_P5IV filtered by logFC ≥ 1. B. Significantly downregulated genes of HALLMARK oxidative phosphorylation of WT31 and WT31_P5IV filtered by logFC ≤ -1. Table 6. A. Significantly upregulated genes of HALLMARK glycolysis of WT31 and WT31_P5IV filtered by logFC ≥ 1. B. Significantly downregulated genes of HALLMARK glycolysis of WT31 and WT31_P5IV filtered by logFC ≤ -1. Table 7. A. Significantly upregulated genes of HALLMARK mitotic spindle of WT31 and WT31_P5IV filtered by logFC ≥ 1. B. Significantly downregulated genes of HALLMARK mitotic spindle of WT31 and WT31_P5IV filtered by logFC ≤ -1. Table 8. A. Significantly upregulated genes of HALLMARK DNA Repair of WT31 and WT31_P5IV filtered by logFC ≥ 1. B. Significantly downregulated genes of HALLMARK DNA-Repair of WT31 and WT31_P5IV filtered by logFC ≤ -1. Table 9. A. Significantly upregulated genes of HALLMARK PI3K/AKT/MTOR-si [file 12885_2023_10912_MOESM2_ESM.pdf]

SUPPLEMENTARY TABLES

Table 1

A. Top twenty-five significantly upregulated genes of RNA Sequencing of WT31 and WT31_P5IV ordered by logFC.

**Gene symbol**	**Gene title**	**LogFC (Fold change) WT31_P5IV > WT31**	**Adjusted p-value**
Mtus2	Microstubule associated tumor suppressor candidate 2	7,889609	3,82 e^{-19}
Gm773	Predicted gene 773	6,258188	3,68 e^{-26}
Gpm6a	Glycoprotein m6a	5,999497	2,59 e^{-13}
Capg	Capping protein (actin filament), gelsolin-like	5,309434	2,35 e^{-20}
Pcdhgb8	Protocadherin gamma subfamily B,8	5,020513	1,85 e^{-11}
Plagl	pleiomorphic adenoma gene-like 1	5,001319	1,7E^{-21}
Nckap1l	NCK associated protein 1 like	4,866565	1,35E^{-11}
Apoe	apolipoprotein E	4,827496	1,72E^{-29}
Mst1r	macrophage stimulating 1 receptor (c-met-related tyrosine kinase)	4,611238	2,01E^{-22}
Cyth4	cytohesin 4	4,569155	8,93E^{-13}
Car12	carbonic anhydrase 12	4,55133	8,4E^{-12}
Acpp	acid phosphatase, prostate	4,352187	1,05E^{-09}
H2-DMb1	histocompatibility 2, class II, locus Mb1	4,344204	4,35E^{-11}
Gstt1	glutathione S-transferase, theta 1	4,343594	3,37E^{-10}
Clstn3	calsyntenin 3	4,267357	3,23E^{-14}
Nxf3	nuclear RNA export factor 3	4,264596	9,04E^{-11}
Col3a1	collagen, type III, alpha 1	4,130455	0,000442
Ephb6	Eph receptor B6	4,108358	1,61E^{-11}
Scml2	Scm polycomb group protein like 2	4,093448	1,69E^{-16}
Tap1	transporter 1, ATP-binding cassette, sub-family B (MDR/TAP)	3,908313	1,86E^{-16}
Pcdhgb1	protocadherin gamma subfamily B, 1	3,817502	1,41E^{-17}
Rhd	Rh blood group, D antigen	3,769838	0,003065
H2-T10	histocompatibility 2, T region locus 10	3,734833	1,97E^{-10}
Hoxa9	homeobox A9	3,718555	5,93E^{-15}

Lrrc8e	leucine rich repeat containing 8 family, member E	3,699302	1,07E^{-13}

B. Top twenty-five significantly downregulated genes of RNA Sequencing of WT31 and WT31_P5IV ordered by logFC.

**Gene symbol**	**Gene title**	**Fold change WT31_P5IV > WT31**	**Adjusted p-value**
Asxl3	Additional sex combs like 3, transcriptional regulator	-8,58037	1,88 e^{-21}
Igfbp3	Insulin-like growth factor binding protein 3	-8,5272	1,78 e^{-21}
Egflam	EGF-like, fibronectin type III and laminin G domains	-8,49434	3,46 e^{-20}
Sox2	SRY (sex determining region Y)-box2	-8,36466	6,13 e^{-20}
Tbc1d9	TBC1 domain family, member 9	-8,0616	6,24 e^{-18}
Pcdh18	protocadherin 18	-7,3263	5,61E^{-13}
Lhfpl3	lipoma HMGIC fusion partner-like 3	-7,08109	6,33E^{-17}
Ngef	neuronal guanine nucleotide exchange factor	-6,99616	1,5E^{-16}
Pcbp3	poly(rC) binding protein 3	-6,96162	1,45E^{-16}
Krt23	keratin 23	-6,81186	9,49E^{-18}
Tbx3	T-box 3	-6,77127	6,85E^{-20}
Ntm	neurotrimin	-6,6698	2,22E^{-13}
Emb	embigin	-6,65169	1,85E^{-15}
Col4a2	collagen, type IV, alpha 2	-6,64165	4,47E^{-19}
Nectin1	nectin cell adhesion molecule 1	-6,3976	8,39E^{-18}
Tenm3	teneurin transmembrane protein 3	-6,24697	3,3E^{-16}
Twist2	twist basic helix-loop-helix transcription factor 2	-6,1489	1,32E^{-18}
Adgrg6	adhesion G protein-coupled receptor G6	-5,87134	4,2E^{-13}
Vat1l	vesicle amine transport protein 1 like	-5,84443	3,27E^{-21}
Tril	TLR4 interactor with leucine-rich repeats	-5,84192	2,7E^{-20}
Ehd3	EH-domain containing 3	-5,7869	9,48E^{-14}
Col4a1	collagen, type IV, alpha 1	-5,73721	1,01E^{-20}
Slc2a13	solute carrier family 2 (facilitated glucose transporter), member 13	-5,67936	1,78E^{-15}

Grik1	glutamate receptor, ionotropic, kainate 1	-5,62	7,34E^{-21}
Gm20619	predicted gene 20619	-5,5811	1,13E^{-14}

Table 2

A. Significantly upregulated genes of GO:0007155 (cell adhesion) of WT31 and WT31_P5IV filtered by logFC ≥ 1.

**Gene symbol**	**Gene title**	**LogFC (Fold change) WT31_P5IV > WT31**	**Adjusted p-value**
Clstn3	calsyntenin 3	4,26459578	3,23 e^{-14}
Pcdhgb1	protocadherin gamma subfamily B, 1	3,76983767	1,41 e^{-17}
Ptk7	PTK7 protein tyrosine kinase 7	3,40372598	2,96 e^{-22}
Pcdha10	protocadherin alpha 10	3,3306971	1,54 e^{-05}
Thbs2	thrombospondin 2	3,15827732	4,67 e^{-06}
Pcdhb4	protocadherin beta 4	2,51612257	8,71 e^{-15}
Msln	mesothelin	2,29389022	5,51 e^{-05}
Pcdhb12	protocadherin beta 12	2,06044067	6,26 e^{-19}
Itga2b	integrin alpha 2b	2,03999075	6,97 e^{-06}
Ache	acetylcholinesterase	2,03219096	1,62 e^{-05}
Pcdhb5	protocadherin beta 5	1,96752745	5,13 e^{-15}
Dchs1	dachsous cadherin related 1	1,79808206	0,01017939
Icam2	intercellular adhesion molecule 2	1,62784551	0,00021795
Trip6	thyroid hormone receptor interactor 6	1,58964485	2,80 e^{-22}
Itgb2	integrin beta 2	1,53208795	0,00057629
Itgal	integrin alpha L	1,46543107	0,00043719
Ccn6	cellular communication network factor 6	1,40473113	0,0001794
Tcam1	testicular cell adhesion molecule 1	1,40047488	1,66 e^{-10}
Tyro3	TYRO3 protein tyrosine kinase 3	1,38540119	4,39 e^{-20}
Itga4	integrin alpha 4	1,32294822	5,64 e^{-17}
Hes1	hes family bHLH transcription factor 1	1,32053009	8,26 e^{-12}
Pcdhb3	protocadherin beta 3	1,29436928	8,87 e^{-12}
Ceacam1	carcinoembryonic antigen-related cell adhesion molecule 1	1,29353265	7,35 e^{-10}
Pcdhb2	protocadherin beta 2	1,25162072	8,82 e^{-13}
Pcdhb8	protocadherin beta 8	1,23851252	3,72 e^{-07}
Pcdhb6	protocadherin beta 6	1,22638958	8,59 e^{-05}

Cntnap4	contactin associated protein-like 4	1,21292404	0,02801693
Cercam	cerebral endothelial cell adhesion molecule	1,2119654	0,0007327
Vwf	Von Willebrand factor	1,20040173	0,00434117
Cdh24	cadherin-like 24	1,18620485	5,77 e^{-16}
Podxl2	podocalyxin-like 2	1,16635324	6,24 e^{-20}
Fbln7	fibulin 7	1,14054038	0,00102016
Ephb4	Eph receptor B4	1,11471526	7,08 e^{-20}
Lama5	laminin, alpha 5	1,11209286	4,86 e^{-14}
Pcdhb11	protocadherin beta 11	1,10199971	2,90 e^{-08}
Ptpru	protein tyrosine phosphatase, receptor type, U	1,0999868	1,03 e^{-06}
Pcdhb16	protocadherin beta 16	1,08971485	3,38 e^{-16}
Nexn	nexilin	1,08148333	5,95 e^{-08}
Sdk1	sidekick cell adhesion molecule 1	1,07739785	1,21 e^{-15}
Tenm2	teneurin transmembrane protein 2	1,07460127	5,53 e^{-13}
Clstn1	calsyntenin 1	1,06642356	1,28 e^{-22}
Fermt3	fermitin family member 3	1,01925766	5,14 e^{-06}
Col18a1	collagen, type XVIII, alpha 1	1,01197122	6,17 e^{-08}

B. Significantly downregulated genes of GO:0007155 (cell adhesion) of WT31 and WT31_P5IV filtered by logFC ≤ -1.

**Gene symbol**	**Gene title**	**LogFC (Fold change) WT31_P5IV < WT31**	**Adjusted p-value**
Pcdh18	protocadherin 18	-7,326296	5,61 e^{-13}
Ntm	neurotrimin	-6,66979817	2,22 e^{-13}
Emb	embigin	-6,65168554	1,85 e^{-15}
Nectin1	nectin cell adhesion molecule 1	-6,39760493	8,39 e^{-18}
Tenm3	teneurin transmembrane protein 3	-6,24697261	3,30 e^{-16}
Spp1	secreted phosphoprotein 1	-4,94892771	3,58 e^{-10}
Ptprf	protein tyrosine phosphatase, receptor type, F	-4,74940646	1,53 e^{-14}
Cd24a	CD24a antigen	-4,47083404	5,07 e^{-07}
Tnc	tenascin C	-4,10792267	1,92 e^{-16}
Cd93	CD93 antigen	-3,64084695	5,39 e^{-07}
Pcdhac2	protocadherin alpha subfamily C, 2	-3,10988809	1,23 e^{-10}
Pcdhga9	protocadherin gamma subfamily A, 9	-2,90739873	1,97 e^{-08}
Cdh11	cadherin 11	-2,68579923	4,63 e^{-19}
Adam23	a disintegrin and metallopeptidase domain 23	-2,56773168	4,24 e^{-16}

Frem2	Fras1 related extracellular matrix protein 2	-2,47034878	1,12 e^{-17}
Hapln1	hyaluronan and proteoglycan link protein 1	-2,41456066	0,00075707
Alcam	activated leukocyte cell adhesion molecule	-2,38571204	1,06 e^{-08}
Cyp1b1	cytochrome P450, family 1, subfamily b, polypeptide 1	-2,25731239	4,14 e^{-05}
Negr1	neuronal growth regulator 1	-2,11717817	0,00036531
Col5a1	collagen, type V, alpha 1	-2,07026326	4,41 e^{-12}
Emp2	epithelial membrane protein 2	-1,97094481	1,36 e^{-08}
Pcdhga7	protocadherin gamma subfamily A, 7	-1,9562213	8,30 e^{-08}
Col28a1	collagen, type XXVIII, alpha 1	-1,90464263	3,29 e^{-06}
Col6a1	collagen, type VI, alpha 1	-1,85329285	0,00033659
Plcb1	phospholipase C, beta 1	-1,82209149	6,57 e^{-19}
Itgb5	integrin beta 5	-1,78156363	2,50 e^{-05}
Ccn4	cellular communication network factor 4	-1,73782445	0,0002341
Igfbp7	insulin-like growth factor binding protein 7	-1,656855	1,08 e^{-14}
Emilin1	elastin microfibril interfacer 1	-1,57169174	4,27 e^{-17}
Kitl	kit ligand	-1,5581631	0,00213062
Ccn2	cellular communication network factor 2	-1,53453531	5,37 e^{-07}
Efnb2	ephrin B2	-1,52835511	2,06 e-10
Cyfip2	cytoplasmic FMR1 interacting protein 2	-1,52020662	1,59 e^{-18}
Chl1	cell adhesion molecule L1-like	-1,48552362	1,42 e^{-13}
Cdh17	cadherin 17	-1,48305335	0,00037236
Pcdh1	protocadherin 1	-1,45356001	2,86 e^{-11}
Pcdhga10	protocadherin gamma subfamily A, 10	-1,42857092	0,04158111
Radil	Ras association and DIL domains	-1,34644525	0,02234839
Pmp22	peripheral myelin protein 22	-1,30087622	0,00153895
Cdh10	cadherin 10	-1,23699451	0,00948591
Flrt3	fibronectin leucine rich transmembrane protein 3	-1,22816475	1,36 e^{-10}
Tgfbi	transforming growth factor, beta induced	-1,12371701	9,62 e^{-07}
Cdh6	cadherin 6	-1,08601511	1,46 e^{-09}
Atp1b1	ATPase, Na+/K+ transporting, beta 1 polypeptide	-1,05373222	0,02311113

Lrfn3	leucine rich repeat and fibronectin type III domain containing 3	-1,04320773	0,00012562
Myh10	myosin, heavy polypeptide 10, non-muscle	-1,04196393	7,06 e^{-16}

Table 3

A. Significantly upregulated genes of GO:0030155 (regulation of cell adhesion) of WT31 and WT31_P5IV filtered by logFC ≥ 1.

**Gene symbol**	**Gene title**	**LogFC (Fold change) WT31_P5IV > WT31**	**Adjusted p-value**
Jag2	jagged 2	1,396515	8,34 e^{-06}
Cxcr3	chemokine (C-X-C motif) receptor 3	1,224663	6,70 e^{-09}
Cytip	cytohesin 1 interacting protein	1,207619	0,013357
Lama5	laminin, alpha 5	1,112093	2,90 e^{-15}

B. Significantly downregulated genes of GO:0030155 (regulation of cell adhesion) of WT31 and WT31_P5IV filtered by logFC ≤ -1.

**Gene symbol**	**Gene title**	**LogFC (Fold change) WT31_P5IV < WT31**	**Adjusted p-value**
Tnc	tenascin C	-4,107922666	1,92 e^{-16}
Sox9	SRY (sex determining region Y)-box 9	-1,03995342	0,0024522

Table 4

A. Significantly upregulated genes of Integrin and disintegrin-like proteins of WT31 and WT31_P5IV filtered by logFC ≥ 1.

**Gene symbol**	**Gene title**	**LogFC (Fold change)**	**Adjusted p-value**

		**WT31_P5IV > WT31**	
Itga10	integrin, alpha 10	2,03999075	6,97 e^{-06}
Itgb1bp2	integrin beta 1 binding protein 2	1,78125759	0,00200661
Itga4	integrin alpha 4	1,53208795	0,00057629
Itgal	integrin alpha L	1,46543107	0,00043719
Itgb2	integrin beta 2	1,32294822	5,64 e^{-17}
Adamts2	a disintegrin-like and metallopeptidase (reprolysin type) with thrombospondin type 1 motif, 2	1,21066482	2,87 e^{-07}
Itga2b	integrin alpha 2b	1,0019518	5,97 e^{-07}

B. Significantly downregulated genes of Integrin and disintegrin-like proteins of WT31 and WT31_P5IV filtered by logFC ≤ -1.

**Gene symbol**	**Gene title**	**LogFC (Fold change) WT31_P5IV < WT31**	**Adjusted p-value**
Adamts18	a disintegrin-like and metallopeptidase (reprolysin type) with thrombospondin type 1 motif, 18	-4,50061089	8,77 e^{-15}
Adam23	a disintegrin and metallopeptidase domain 23	-2,56773168	4,24 e^{-16}
Itgb5	integrin beta 5	-1,78156363	2,50 e^{-05}
Adamts8	a disintegrin-like and metallopeptidase (reprolysin type) with thrombospondin type 1 motif, 8	-1,68481531	0,0011165

Table 5

A. Significantly upregulated genes of HALLMARK oxidative phosphorylation of WT31 and WT31_P5IV filtered by logFC ≥ 1.

**Gene symbol**	**Gene title**	**LogFC (Fold change) WT31_P5IV > WT31**	**Adjusted p-value**
Retsat	retinol saturase (all trans retinol 13,14 reductase)	1,474494485	3,91 e^{-23}

B. Significantly downregulated genes of HALLMARK oxidative phosphorylation of WT31 and WT31_P5IV filtered by logFC ≤ -1.

Gene symbol	Gene title	LogFC (Fold change) WT31_P5IV < WT31	Adjusted p-value
Phyh	phytanoyl-CoA hydroxylase	-1,32445051	4,06 e^{-10}
Bdh2	3-hydroxybutyrate dehydrogenase, type 2	-1,27189857	2,70 e^{-09}
Atp1b1	ATPase, Na+/K+ transporting, beta 1 polypeptide	-1,05373222	0,02311113
Prdx3	peroxiredoxin 3	-1,01917925	1,01 e^{-20}
Maob	monoamine oxidase B	-1,00913638	0,01828803

Table 6

A. Significantly upregulated genes of HALLMARK glycolysis of WT31 and WT31_P5IV filtered by logFC ≥ 1.

Gene symbol	Gene title	LogFC (Fold change) WT31_P5IV > WT31	Adjusted p-value
B3gnt3	UDP-GlcNAc:betaGal beta-1,3-N-acetylglucosaminyltransferase 3	1,43266644	4,99 e^{-07}
Spag4	sperm associated antigen 4	1,40399594	1,37 e^{-09}
B4galt2	UDP-Gal:betaGlcNAc beta 1,4- galactosyltransferase, polypeptide 2	1,24302768	0,0004979
Agrn	agrin	1,03369579	8,60 e^{-19}
Abcb6	ATP-binding cassette, sub-family B (MDR/TAP), member 6	1,02951407	1,82 e^{-20}
Pkp2	plakophilin 2	1,02069753	0,00071865
Xylt2	xylosyltransferase II	1,01948689	3,82 e^{-19}

B. Significantly downregulated genes of HALLMARK glycolysis of WT31 and WT31_P5IV filtered by logFC ≤ -1.

Gene symbol	Gene title	LogFC (Fold change) WT31_P5IV < WT31	Adjusted p-value
Igfbp3	insulin-like growth factor binding protein 3	-8,52719653	1,78 e^{-21}
Hs6st2	heparan sulfate 6-O-sulfotransferase 2	-4,88590316	3,59 e^{-16}
Stc2	stanniocalcin 2	-3,65937514	8,87 e^{-12}

Slc25a13	solute carrier family 25 (mitochondrial carrier, adenine nucleotide translocator), member 13	-3,62598702	$7,87\ e^{-19}$
Col5a1	collagen, type V, alpha 1	-2,07026326	$4,41\ e^{-12}$
Chst2	carbohydrate sulfotransferase 2	-2,03386691	$5,18\ e^{-25}$
Tgfbi	transforming growth factor, beta induced	-1,12371701	$9,62\ e^{-07}$
Sox9	SRY (sex determining region Y)-box 9	-1,03995342	0,0024522
Me1	malic enzyme 1, NADP(+)-dependent, cytosolic	-1,02274342	$3,71\ e^{-17}$

Table 7

A. Significantly upregulated genes of HALLMARK mitotic spindle of WT31 and WT31_P5IV filtered by logFC ≥ 1.

**Gene symbol**	**Gene title**	**LogFC (Fold change) WT31_P5IV > WT31**	**Adjusted p-value**
Fscn1	fascin actin-bundling protein 1	1,95044031	0,00222569
Sun2	Sad1 and UNC84 domain containing 2	1,21674902	$3,59\ e^{-17}$
Notch2	notch 2	1,11989632	$2,35\ e^{-21}$

B. Significantly downregulated genes of HALLMARK mitotic spindle of WT31 and WT31_P5IV filtered by logFC ≤ -1.

**Gene symbol**	**Gene title**	**LogFC (Fold change) WT31_P5IV < WT31**	**Adjusted p-value**
Sorbs2	sorbin and SH3 domain containing 2	-4,24747014	$1,57\ e^{-13}$
Arhgef3	Rho guanine nucleotide exchange factor (GEF) 3	-4,14742954	$7,18\ e^{-15}$
Myh10	myosin, heavy polypeptide 10, non-muscle	-1,04196393	$2,44\ e^{-17}$

Table 8

A. Significantly upregulated genes of HALLMARK DNA Repair of WT31 and WT31_P5IV filtered by logFC ≥ 1.

**Gene symbol**	**Gene title**	**LogFC (Fold change) WT31_P5IV > WT31**	**Adjusted p-value**
Npr2	natriuretic peptide receptor 2	1,657901682	3,74 e^{-18}
Pde6g	phosphodiesterase 6G, cGMP-specific, rod, gamma	1,358388074	0,000132886

B. Significantly downregulated genes of HALLMARK DNA-Repair of WT31 and WT31_P5IV filtered by logFC ≤ -1.

**Gene symbol**	**Gene title**	**LogFC (Fold change) WT31_P5IV < WT31**	**Adjusted p-value**
Ago4	argonaute RISC catalytic subunit 4	-1,755334623	0,000880622

Table 9

A. Significantly upregulated genes of HALLMARK PI3K/AKT/MTOR-signaling of WT31 and WT31_P5IV filtered by logFC ≥ 1.

**Gene symbol**	**Gene title**	**LogFC (Fold change) WT31_P5IV > WT31**	**Adjusted p-value**
Pitx2	paired-like homeodomain transcription factor 2	2,631017022	1,51 e^{-06}
Lck	lymphocyte protein tyrosine kinase	1,676031417	0,00206918
Nod1	nucleotide-binding oligomerization domain containing 1	1,138919123	7,64 e^{-07}

B. Significantly downregulated genes of HALLMARK PI3K/AKT/MTOR-signaling of WT31 and WT31_P5IV filtered by logFC ≤ -1.

**Gene symbol**	**Gene title**	**LogFC (Fold change) WT31_P5IV < WT31**	**Adjusted p-value**
Adcy2	adenylate cyclase 2	-5,173804043	2,69 e^{-19}
Vav3	vav 3 oncogene	-3,517678911	1,41 e^{-06}
Plcb1	phospholipase C, beta 1	-1,822091492	6,57 e^{-19}

Prkcb	protein kinase C, beta	-1,6605389	0,000412393

Table 10

A. Primers used for qRT-PCR

**Primer for qRT-PCR**	**Sequence**	**Supplier**
Mm_Capg_F	TGCAGCCCTGTATAAGGTCTCTG	Metabion International AG
Mm_Capg_R	AGATGAAGCCATCAGCCACTT	Metabion International AG
Mm_Gm771_F	AACTTGTAAGCCTGTTTCACCCAA	Metabion International AG
Mm_Gm771_R	AAATACTAGGCTTCTTGCTGTAAGT	Metabion International AG
Mm_Gpm6a_F	CTAAGCTCCAAGGACTTGCC	Metabion International AG
Mm_Gpm6a_R	CACTCGAAGCACCCTTTCTGT	Metabion International AG
Mm_Pcdhgb8_F	CGCGAGACCTTTGTACGGA	Metabion International AG
Mm_Pcdhgb8_R	GCGGGGCTTGGTGATTAGAA	Metabion International AG
Mm_Igfbp3_F	AGATGCTCCGTGCCACATAA	Metabion International AG
Mm_Igfbp3_R	GCTGTAGCCAGCTGCTGAT	Metabion International AG
Mm_Notch2_F	ATGCCGTGGGGCTGAAAAAT	Metabion International AG
Mm_Notch2_R	TTCCGACAGCAAAGCCTCAT	Metabion International AG
Mm_Sun2_F	CTGGAATCAGGAGGAGCCAG	Metabion International AG
Mm_Sun2_R	ACGTGCTCTAAGGTAACGGC	Metabion International AG
Mm_Arhgef3_F	CAAGAGACAGGAGGCGATCTT	Metabion International AG
Mm_Arhgef3_R	CTCGAAGCTGACTGAGGAGC	Metabion International AG
Mm_Sorbs2_F	ACGGATGGTTTGTGGGAACT	Metabion International AG
Mm_Sorbs2_R	CCTCCTGCTGGTGATTTGGT	Metabion International AG
Mm_Itga2b_F	GCCATCTCCCAAGGTGGAC	Metabion International AG
Mm_Itga2b_R	TCACACTCCACCACCGTACA	Metabion International AG
Mm_Itga4_F	CAGCAAAAAGGCATAGCGGG	Metabion International AG
Mm_Itga4_R	CAGCTTTCATGCAATACAGGAGT	Metabion International AG

Mm_Adam23_F	TGATTTCACTTGGGCAGGCA	Metabion International AG
Mm_Adam23_R	TGGTGGCGCTAGGACCCTTA	Metabion International AG
Mm_Itgb5_F	GCCCGTTATGAAATGGCCTC	Metabion International AG
Mm_Itgb5_R	AGCTAGCGTGAGCAAATGGT	Metabion International AG
Mm_Adamts18_F	CTCCTGAAAAGAAAGATGACCCA	Metabion International AG
Mm_Adamts18_R	GAGGAGACCCCAAGATCAGC	Metabion International AG
Mm_Gak_F	CTGCCCACCAGGCATTTG	Metabion International AG
Mm_Gak_R	CCATGTCACATACATATTCAATGTACCT	Metabion International AG
Mm_Srp72_F	CACCCAGCAGACAGACAAACTG	Metabion International AG
Mm_Srp72_R	GCACTCATGGTAGCGTTCCA	Metabion International AG
